# SGK2 promotes prostate cancer metastasis by inhibiting ferroptosis via upregulating GPX4

**DOI:** 10.1038/s41419-023-05614-5

**Published:** 2023-01-31

**Authors:** Lulin Cheng, Qingliu He, Bing Liu, Liang Chen, Fang Lv, Xuexiang Li, Yunxue Li, Chunyu Liu, Yarong Song, Yifei Xing

**Affiliations:** Department of Urology, Union Hospital, Tongji Medical College, Huazhong University of Science and Technology, 430022 Wuhan, China

**Keywords:** Prostate cancer, Cell invasion

## Abstract

Recent research has shown that ferroptosis, the iron-dependent accumulation of lipid peroxides that leads to cell death, suppresses cancer metastasis. However, the role of ferroptosis in prostate cancer metastasis has not been completely elucidated. In the current study, we identified the essential role of serum/glucocorticoid regulated kinase 2 (SGK2) in promoting prostate cancer metastasis by inhibiting ferroptosis. We found that the expression of SGK2 was higher in metastatic prostate cancer and predicted poor clinical outcomes. SGK2 knockdown inhibited the metastatic capacity of prostate cancer cells in vivo and in vitro, while SGK2 overexpression inhibited ferroptosis and facilitated prostate cancer metastasis by phosphorylating the Thr-24 and Ser-319 sites of forkhead box O1 (FOXO1). This process induced the translocation of FOXO1 from the nucleus to the cytoplasm, relieving the inhibitory effect of FOXO1 on glutathione peroxidase 4 (GPX4). These findings delineated a novel role of SGK2 in ferroptosis regulation of prostate cancer metastasis, identifying a new key pathway driving prostate cancer metastasis and potentially providing new treatment strategies for metastatic prostate cancer.

## Introduction

Prostate cancer (PCa) is the second most frequently diagnosed cancer among men worldwide [1]. Although the rate of early detection of PCa has increased as a result of prostate-specific antigen screening [2, 3], cases of metastasis are identified at first diagnosis, even now [4, 5]. PCa cells often spread to lymph nodes, bones, and other sites [6]. Patients with metastatic PCa will eventually be insensitive to antitumor treatment and demonstrate high frequencies of post-treatment relapse. Metastasis is one of the leading causes of PCa-related deaths. Hence, there is an urgent need to explore relevant metastatic mechanisms of PCa to obtain more effective antitumor treatments.

By mining the Cancer Genome Atlas (TCGA) and Gene-Expression Omnibus (GEO) datasets, we identified that serum/glucocorticoid regulated kinase 2 (SGK2) was associated with PCa metastasis. SGK2 belongs to the SGK family of AGC kinases, which includes SGK1, SGK2, and SGK3 gene [7]. SGK family mainly regulate ion channels, transport, hormone release, neuro-excitability, and transcription factors [8]. Literature shows that most scientific knowledge about the role of SGK proteins in human pathophysiology is based on SGK1 and SGK3 research, with relatively little research regarding SGK2. Recent reports have demonstrated that SGK2 upregulation promotes the progression of metastasis in bladder, kidney, and colon cancers [9–11], but the relationship between SGK2 and PCa metastasis is unclear.

Ferroptosis is a newly discovered form of iron-dependent cell death, characterized by the accumulation of lipid peroxidation, which differs from apoptosis, necrosis, and autophagy [12, 13]. Recently, resistance to ferroptosis has been found to be an important feature of breast cancer metastasis [14]. Neratinib inhibited metastasis by promoting ferroptosis in breast cancer cells [15]. In addition, deletion of KLF2 significantly inhibited ferroptosis and improved the invasion of clear cell renal cell carcinoma [16]. However, there are few studies on the relationship between PCa metastasis and ferroptosis, and it is crucial to explore whether ferroptosis regulation can inhibit PCa metastasis.

In this study, we found that SGK2 promoted PCa metastasis by inhibiting ferroptosis, which was previously unrecognized.

## Materials and methods

### Reagents

Erastin (HY-15763) and Z-VAD-FMK (HY-16658B) were purchased from MedChemExpress (Shanghai, China). Ferrostain-1 (S7243) and 3-Methyladenine (S2767) were purchased from Selleck Chemical (Shanghai, China).

### Cell lines and cell culture

The PC3 and DU145 PCa cell lines were purchased from the Cell Bank of the Chinese Academy of Sciences (Shanghai, China). All cells were cultured in RPMI 1640 medium (Hyclone, GE Healthcare Life Sciences, Logan, UT, USA) supplemented with 10% FBS (Biological Industries) and 1% penicillin/streptomycin (Beyotime Institute of Biotechnology, Nanjing, China) at 37 °C with 5% CO_2_.

### Patient tissue specimens

In this study, 40 sets of PCa tissue samples and their corresponding lymph node metastasis tissues were obtained from patients who underwent radical prostatectomy with extended pelvic lymph node dissection for PCa at the Department of Urology of Union Hospital affiliated with Tongji Medical College between 2016 and 2021. All patients signed informed consent forms. All procedures were in accordance with Declaration of Helsinki and the research protocols were approved by the Research Ethics Committees of Union Hospital, Tongji Medical College, and Huazhong University of Science and Technology.

### Western blotting

RIPA lysis buffer (Servicebio, Wuhan, China) was used to extract total protein from the cells. A BCA Protein Assay Kit (Beyotime Biotechnology, Shanghai, China) was used to determine the protein concentration. Sodium dodecyl sulfate- polyacrylamide gel electrophoresis (SDS-PAGE) was used to separate out equal amounts of the protein, which was then transferred to a polyvinylidene difluoride (PVDF) membrane (Millipore, Eschborn, Germany). Next, 5% skimmed milk was used as a blocking agent on the PVDF membrane for 1 h. The membrane was washed with 1X Tris-buffered saline with 0.05% Tween 20 (TBST) three times for 10 min each. The Membrane was then incubated with primary antibodies overnight at 4 °C, followed by incubation with the corresponding secondary antibodies (HRP conjugated Affinipure Goat anti-mouse antibody, SA00001-1 and HRP conjugated Affinipure Goat anti-rabbit antibody, SA00001-2; ProteinTech) at room temperature (RT) for 1 h. An enhanced chemiluminescence reagent (Servicebio, Wuhan, China) was used to visualize the protein bands. The following primary antibodies were used: SGK2 (5595, Cell Signaling Technology), GPX4 (67763-1-Ig, Proteintech), SLC3A2 (15193-1-AP, Proteintech), AIFM2 (20886-1-AP, Proteintech), SLC7A11 (12691, Cell Signaling Technology), ACSL4 (22401-1-AP, Proteintech), DHODH (14877-1-AP, Proteintech), GAPDH (10494-1-AP, Proteintech), α-Tubulin (66031-1-Ig, Proteintech), Histone-H3 (17168-1-AP, Proteintech), FOXO1 (66457-1-Ig, Proteintech), FOXO3 (10849-1-AP, Proteintech), FOXO4 (21535-1-AP, Proteintech), FOXO6 (19122-1-AP, Proteintech), Phospho-FOXO1 (Ser-256) (ab131339, Abcam), Phospho-FOXO1 (Ser-319) (ab47326, Abcam), and Phospho-FOXO1 (Thr-24) (9464, Cell Signaling Technology).

### Quantitative real-time PCR (qRT-PCR)

The TRIzol reagent (Invitrogen, Carlsbad, CA, USA) was applied to extract total RNA. The iScript cDNA synthesis kit (Bio-Rad, Hercules, CA, USA) was used to synthesize cDNA from total RNA. According to manufacturer’s instructions, the qRT-PCR was performed by using the ChamQ SYBR qPCR Master Mix (Vazyme, Nanjing, China) on a StepOne Plus real-time PCR system (Life Technologies, CA, USA). ACTB was used as internal control. Primer sequences for the study were presented in Supplementary Table. S1.

### Immunohistochemistry (IHC)

Using previously described methods [17], IHC staining was performed with SGK2 (5595, Cell Signaling Technology) and GPX4 (67763-1-Ig, Proteintech) primary antibodies. Subsequently, using a combination of the percentage and intensity of PCa cells that were positively stained, an H-score was generated to assess immunoreactivity. The staining intensity was classified into four grades as follows: (0) no staining; (1) weak staining; (2) moderate staining; and (3) strong staining. H-scores were calculated using the following formula: (percentage of weak ×1) + (percentage of moderate ×2) + (percentage of strong × 3). H-scores ranged from 0–300.

### Cell migration and invasion assays

The migration and invasion abilities of cells were evaluated by applying 8.0 μm pore polycarbonate membrane inserts to 24-well Transwell plates (Corning, NY, USA). For the migration assay, 600 μl complete medium was added to the lower chambers after pre-treatment. Thereafter, cell numbers were counted. Approximately 4 × 10^4^ cells in 200 μl serum-free medium were added to the upper chambers. Cells were cultured at 37 °C in 5% CO_2_ for 48 h. A moistened cotton swab was used to gently wipe off the cells left in the upper chambers. The migratory cells were fixed with 4% paraformaldehyde for 10 min. Migratory cells were stained using 0.1% crystal violet for 30 min at RT. Migratory cells were washed with phosphate-buffered saline (PBS) three times and counted in three randomly selected regions under a 200 × inverted phase-contrast microscope (Olympus, Tokyo, Japan). The invasion assay was performed following the methodology used for the migration assay, except that 50 μl Matrigel matrix (BD Biosciences) was added to each upper chamber.

### Cell viability assay

A cell counting kit-8 (CCK-8) assay (Dojindo Laboratories, Kumamoto, Japan) was used to determine cell viability. The cell suspension was prepared with complete medium and inoculated into 96-well plates (5000 cells/well), with 200 μl cell suspension per well. After conventional culture for 24 h, the CCK-8 solution was added to each well and incubated at 37 °C for 1 h. Finally, absorbance was measured at 450 nm using a microplate reader (Tecan, Mannedorf, Switzerland).

### Intracellular reactive-oxygen species (ROS) detection

A ROS assay kit (Beyotime Biotechnology) was used to detect intracellular ROS levels. The prepared 2ʹ,7ʹ-dichlorofluorescin diacetate (DCFH-DA) diluent was added to cells seeded into 6-well plates and incubated for 30 min at 37 °C in the dark. The cells were then washed thrice with PBS and digested with trypsin. Cell precipitates were collected by centrifugation and resuspended in PBS. Finally, ROS levels were analyzed using a flow cytometer (Beckman Coulter, Indianapolis, IN, USA).

### Malondialdehyde (MDA) detection

MDA is a reliable biomarker of lipid peroxidation. An MDA assay kit (Nanjing Jincheng Bioengineering Institute, China) was used to detect intracellular MDA levels. Briefly, thiobarbituric acid (TBA) reacted with MDA under acidic conditions at 95 °C for 40 min, forming a pink MDA-TBA conjugate. Absorbance was measured at a wavelength of 450 nm. MDA measurements were expressed as nmol/mg cellular protein.

### Measurement of intracellular Fe^2+^

The FeRhoNox™-1 fluorescent probe (GORYO Chemical, Sapporo, Japan) was used to detect intracellular Fe^2+^ levels. Cells were seeded on coverslips for 24 h, then cell culture medium was removed and cells were washed twice with PBS. The 5 μM FeRhoNox™-1 solution was added and cells was incubated for 1 h at 37 °C in the dark. Cells were washed three times with PBS. The nuclei were stained with DAPI for 15 min. Finally, cells were observed by using a Nikon A1Si laser scanning confocal microscope (Nikon Instruments Inc., Japan).

### Detection of mitochondrial membrane potential

The mitochondrial membrane potential assay kit with JC-1(Beyotime Biotechnology) was used to detect the mitochondrial membrane potential according to the manufacturer’s instructions. In cells with high mitochondrial membrane potential, JC-1 accumulated in the matrix of mitochondria and formed J-aggregates, which produced red fluorescence. In cells with low mitochondrial membrane potential, JC-1 did not accumulate in the matrix of mitochondria, and JC-1 was a monomer and produced green fluorescence. The relative ratio of red and green fluorescence was used to measure the levels of mitochondrial membrane potential. The levels of mitochondrial membrane potential were analyzed using a flow cytometer (Beckman Coulter, Indianapolis, IN, USA).

### Transmission electron microscopy (TEM)

After pre-treatment, cells were collected, precipitated, and fixed with 2.5% glutaraldehyde for 2 h at 4 °C. TEM imaging was performed by Servicebio (Wuhan, China).

### Light microscopy

After pre-treatment, an inverted phase-contrast microscope (Olympus) was used to obtain phase- contrast images of cells at 200 × magnification.

### Plasmid transfection

Two types of plasmids were constructed using Vigene Biosciences (Shandong, China); plasmids for SGK2 overexpression and knockdown. In addition, seven types of plasmids were constructed by GeneChem (Shanghai, China), including GPX4 overexpression, FOXO1 overexpression, FOXO1-T24A mutant, FOXO1-S319A mutant, FOXO1-Both (T24A and S319A) mutant, FOXO1 knockdown, and FOXO4 overexpression plasmids. Plasmids and vectors were transfected into PCa cells using Lipofectamine (Invitrogen) according to the manufacturer’s instructions.

### Immunofluorescence staining

Cells were seeded on coverslips for 12 h, then collected and fixed with 4% paraformaldehyde for 10 min. Cells were permeabilized with 0.3% Triton X-100 for 20 min at RT. The cells were blocked using 3% bovine serum albumin for 30 min at RT. Cells were incubated with the primary antibodies overnight at 4 °C. On the second day, the cells were incubated with the corresponding fluorescent-labeled secondary antibodies (Fluorescein-conjugated Affinipure Goat Anti-Rabbit IgG(H + L), SA00003-2, ProteinTech; Fluorescein-conjugated Affinipure Goat Anti-Mouse IgG(H + L), SA00003-1, ProteinTech; Cy3–conjugated Affinipure Goat Anti-Rabbit IgG(H + L), SA00009-2, ProteinTech) for 1 h in the dark. Cells were washed with PBS three times for 10 min each. The nuclei were stained with DAPI for 15 min. Finally, fluorescence images of the cells were obtained using a Nikon A1Si laser scanning confocal microscope (Nikon Instruments Inc., Japan).

### Nuclear and cytosolic protein extraction

A nuclear and cytoplasmic protein extraction kit (Beyotime Biotechnology) was used to separate the nuclear and cytoplasmic portions of the cells. Western blotting was performed to assess extracted proteins. Histone-H3 and α-tubulin were used as internal reference controls for the nuclear and cytoplasmic fractions.

### Co-immunoprecipitation (Co-IP)

Cell precipitates were collected by centrifugation. The cells were resuspended in lysis buffer containing a protease inhibitor cocktail. The cells were then lysed on ice for 30 min, centrifuged at 15,000 rpm for 15 min, and supernatants were collected. Antibodies (5 μg) and proteins (500 μg) were used for immunoprecipitation. The BeyoMag™ Protein A + G Magnetic Beads (Beyotime Biotechnology) were used to collect the precipitated proteins overnight at 4 °C. The magnetic beads were washed with Tris-buffered saline three times. Elution of products was performed by boiling the magnetic beads in Laemmli sample buffer. Finally, Co-IP products and their corresponding inputs were subjected to western blotting. The following antibodies were used: SGK2 (5595, Cell Signaling Technology), FOXO1 (66457-1-Ig, Proteintech), anti-Mouse IgG (ab37355, Abcam) and anti-Rabbit IgG (ab171870, Abcam).

### Luciferase reporter assay

The luciferase reporter assay was used to detect the transcriptional regulation of FOXO1/FOXO4 by GPX4. The three plasmids were co-transfected into PC3 cells. These plasmids were FOXO1/FOXO4 overexpression plasmids and pGL3-basic luciferase reporter plasmids containing the GPX4 promoter and pRL-TK Renilla luciferase reporter vectors. Next, to test the specific binding sites of FOXO1 and GPX4, wild-type/mutant plasmids containing the promoter of GPX4 and plasmids overexpressing FOXO1 were used in PC3 cells. The cells were then cultured for 48 h. The Dual- Luciferase Reporter Assay System (Promega, USA) was used to measure the activity of firefly luciferase and Renilla luciferase.

### Animal experiments

Four-week-old male athymic BALB/c nude mice were purchased from Beijing Vital River Laboratory Animal Technology Co., Ltd. All animal experiments were carried out in accordance with the ARRIVE guidelines. The mice were randomly divided into four groups (five mice per group). For animal experiments, no blinding was performed. PC3 cells were stably labeled with cy3. To establish a model of lung metastasis, we injected PC3 cells (2 × 10^6^) stably transfected with plasmids into the tail vein of each mouse. After 60 days, all mice were sacrificed. Finally, fluorescence images of the xenografts in nude mice were obtained using In Vivo FX PRO (BRUKER Corporation, USA).

### Statistical analysis

Prism 8.0.1 (GraphPad) was used to analyze all the data. All data are presented as mean values with standard deviation (SD). The two-tailed Student’s t-test was performed to compare the differences between two groups. Analysis of variance (ANOVA) was used to analyze the differences among multiple sets of data. Kaplan–Meier analysis was used to verify survival information, and the log-rank test was used to compare differences in survival information. ImageJ software was used to detect the gray value of protein expression. All in vitro experiments were repeated three times. Data were considered statistically significant when the p-value was less than 0.05.

## Results

### The identification of SGK2 promoting PCa metastasis

**Fig. 1:**
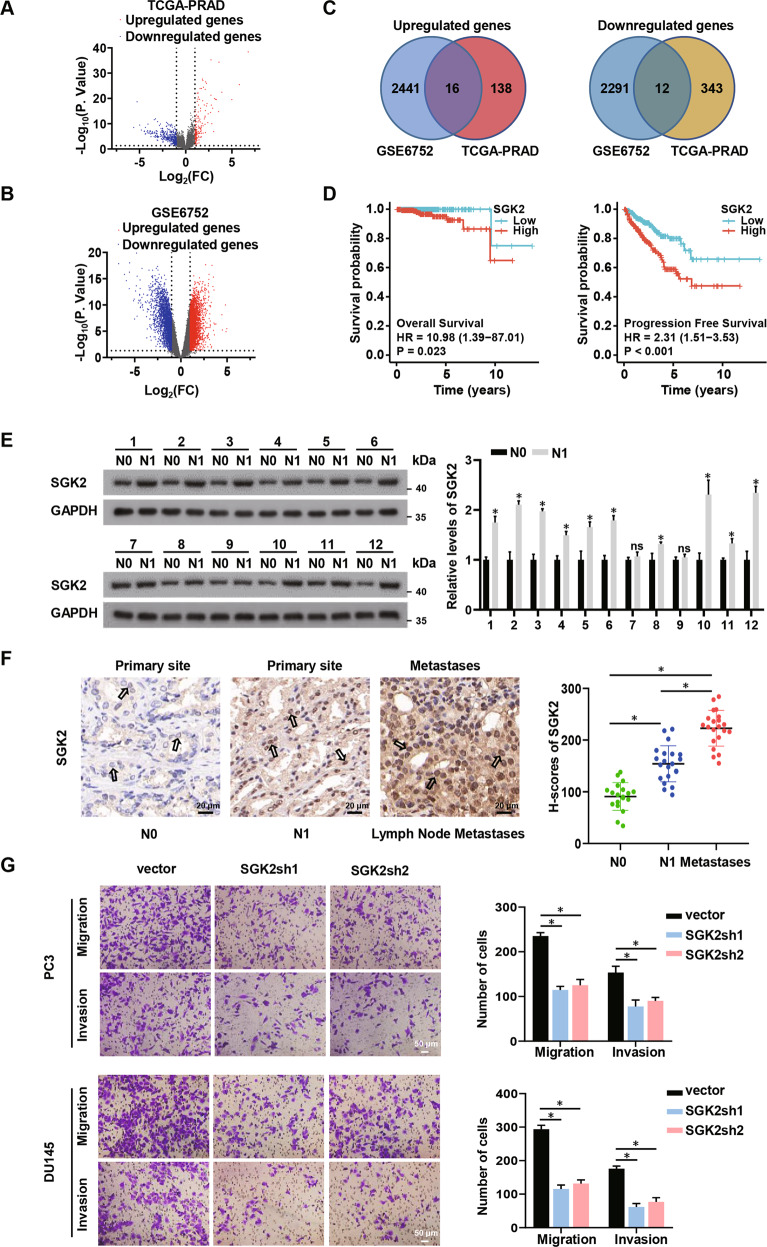
The identification of SGK2 promoting PCa metastasis. **A**, **B** Volcano plots showing differential expressed genes (DEGs) of the TCGA-PRAD group dataset and the GSE6752 dataset. (PRAD, prostate adenocarcinoma). **C** Venn-diagram analysis of upregulated/ downregulated genes in the TCGA-PRAD group dataset and the GSE6752 dataset. **D** OS, PFS were analyzed according to high or low expression of SGK2 from the TCGA database. The number of patients in low-expression groups are 249, and the number of patients in high expression groups are 250. **E** Western blotting analysis of SGK2 expression in PCa tissues without lymph node metastasis(N0) and PCa tissues with lymph node metastasis(N1). **F** Immunohistochemistry (IHC) analysis of SGK2 expression difference and H-scores of SGK2 among PCa tissues without lymph node metastasis(N0) and PCa tissues with lymph node metastasis(N1) and corresponding Lymph Node Metastases tissues. Scale bars, 20 μm. **G** Transwell analysis of the migration and invasion capability of PCa cells with SGK2 knockdown. Scale bars, 50 μm. Data are presented as representative images or as the mean ± SD of three independent experiments. *P < 0.05; ns, not significant.

To identify potential genes that may affect PCa progression, we analyzed TCGA datasets and obtained differentially expressed genes (DEGs) from PCa samples with lymph node metastasis (LNM) and without lymph node metastasis (Fig. 1A; Supplementary Tables S2 and S3). In addition, we acquired DEGs from PCa tissues with or without metastasis by mining GEO datasets (GEO accession: GSE6752) [18] (Fig. 1B; Supplementary Tables S4 and S5). A two-way Venn-diagram demonstrated 16 upregulated candidate genes and 12 downregulated candidate genes in metastatic PCa (|Log_2_FC | > 1, *p* < 0.05) (Fig. 1C). Next, we analyzed the survival and prognosis of these candidate genes in PCa via TCGA datasets. The results showed that only SGK2, one of upregulated candidate genes, was closely related to both worse overall survival (OS) and worse progression free survival (PFS) (Fig. 1D; Supplementary Figs. S1A and S2A).

To verify the expression of SGK2 in metastatic PCa, we collected 21 PCa tissues with LNM and 19 PCa tissues without LNM from our center. The expression levels of SGK2 were significantly elevated in PCa with LNM compared with PCa without LNM, as determined by western blot analysis (Fig. 1E). Consistently, IHC staining for SGK2 showed a similar trend. Moreover, SGK2 was highly expressed in corresponding lymph node metastases (Fig. 1F). In our study, the metastatic cell line PC3 from bone metastasis of PCa and DU145 cells from a human prostatic adenocarcinoma metastatic to the brain were used because of their high invasiveness [19–22]. We established stable PC3 and DU145 cells of SGK2 overexpression and knockdown (Supplementary Fig. S3A). The results of the Transwell assay showed that SGK2 decline significantly reduced migration and invasion abilities compared with that of parental PCa cells (Fig. 1G). However, the opposite results were observed when SGK2 was overexpressed (Supplementary Fig. S3B). Taken together, these results demonstrated that SGK2 expression was higher in metastatic PCa with poor outcomes, and SGK2 upregulation increased the metastatic capacity of PCa cells.

### SGK2 is a key negative regulator of ferroptosis in PCa

**Fig. 2:**
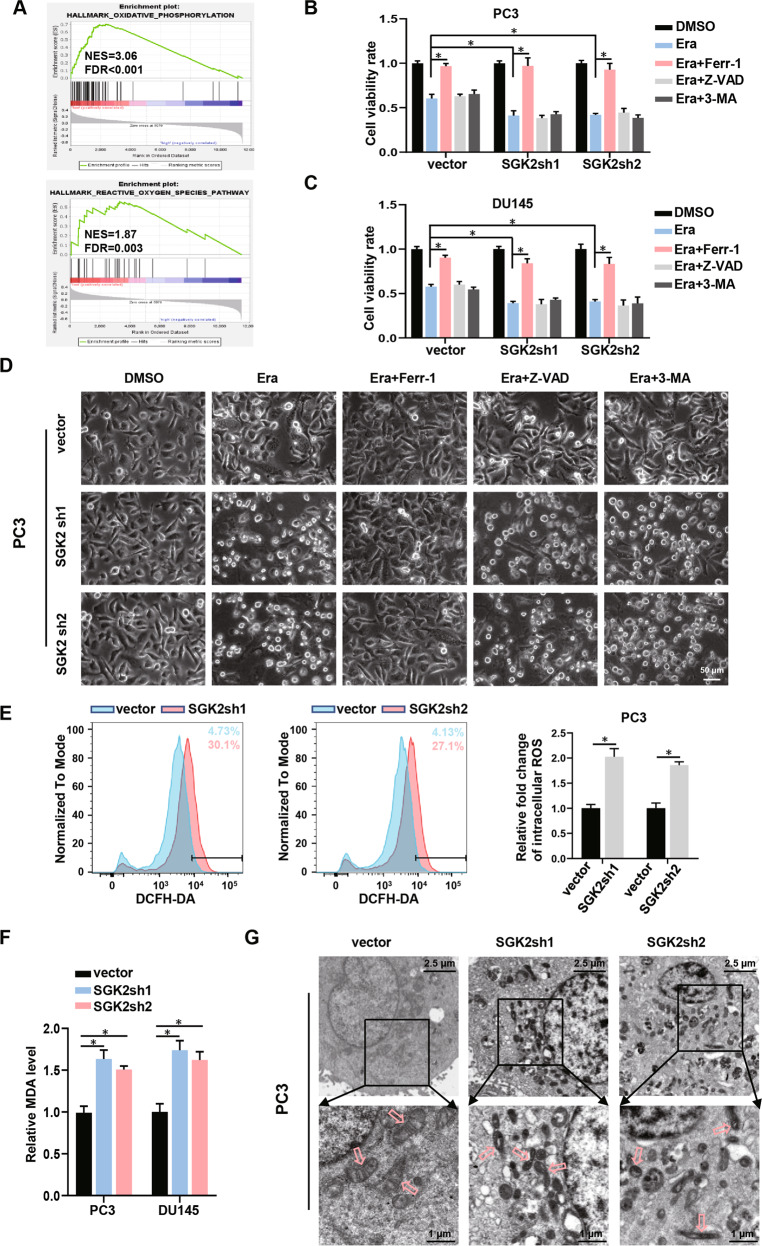
SGK2 is a key negative regulator of ferroptosis in PCa. **A** GSEA enrichment plots by SGK2 high- and low-expression groups for two gene sets from the hallmark database associated with reactive oxygen species and oxidative phosphorylation are shown. **B**, **C** Bar graphs showing cell viability in SGK2 knockdown PCa cells treated with erastin (Era, 5 μM), erastin (Era, 5 μM) combined with Ferrostain-1(Ferr-1, 5 μM), Z-VAD-FMK (Z-VAD, 20 μM) or 3-Methyladenine (3-MA, 10 mM) for 24 h, respectively. **D** Representative phase-contrast images of PC3 cells with SGK2 knockdown treated with Era (5 μM), Era (5 μM) combined with Ferr-1(5 μM), Z-VAD (20 μM), or 3-MA (10 mM) for 24 h, respectively. Scale bars, 50 µm. **E** Intracellular ROS of PC3 cells with SGK2 knockdown was stained by DCFH-DA and determined by flow cytometry. **F** Concentrations of MDA were measured in PCa cells with SGK2 knockdown. **G** The morphological changes of mitochondria were detected by TEM in PC3 cells with SGK2 knockdown. Scale bars represent 2.5 µm and 1 µm, respectively. Data are presented as representative images or as the mean ± SD of three independent experiments. *P < 0.05.

To explore the potential mechanism of SGK2 increase in enhancing aggressiveness in PCa, we performed Gene Set Enrichment Analysis (GSEA) to analyze the altered gene sets in the high and low-SGK2 gene-expression groups. Gene-expression data for SGK2 were obtained from TCGA database. Previous studies have shown that the decrease of oxidative phosphorylation and ROS levels inhibit ferroptosis [23, 24]. Recent studies have reported that inhibiting ferroptosis promotes the metastasis of breast cancer, clear cell renal cell carcinoma, and melanoma [14–16, 25]; therefore, we presumed that the increase of SGK2 in facilitating PCa metastasis may be mediated by inhibiting ferroptosis (Fig. 2A). To study the relationship between SGK2 and ferroptosis, we first performed the CCK-8 assay and observed changes in cell morphology after various treatments. As shown in Fig. 2B and D, stable knockdown of SGK2 markedly accelerated the increase in erastin-induced cell death in PC3 cells, but this effect was blocked by ferrostatin-1 instead of the apoptosis inhibitor (Z-VAD-FMK) and the autophagy inhibitor (3-Methyladenine). Similar effects were observed in DU145 cells (Fig. 2C; Supplementary Fig. S4A). We also found that ROS and MDA levels were significantly elevated by SGK2 downregulation in PCa cells. (Fig. 2E, F; Supplementary Fig. S4B). In addition, the opposite effect was observed in PCa cells stably overexpressing SGK2 (Supplementary Fig. S4C–F). Consistent with these results, the morphological characteristics of typical ferroptosis were observed using transmission electron microscopy in SGK2 knockdown PCa cells, including mitochondrial atrophy, shrinkage, and increased membrane density (Fig. 2G; Supplementary Fig. S4G). The mitochondrial membrane potential levels were significantly decreased by SGK2 downregulation in PCa cells (Supplementary Fig. S5A, B). Intracellular Fe^2+^ ion levels were not significant difference when SGK2 was overexpressed or knocked down compared with the control group. (Supplementary Fig. S5C, D). These results indicated that SGK2 downregulation promoted ferroptosis in PCa.

### SGK2 knockdown facilitates ferroptosis by downregulating GPX4

**Fig. 3:**
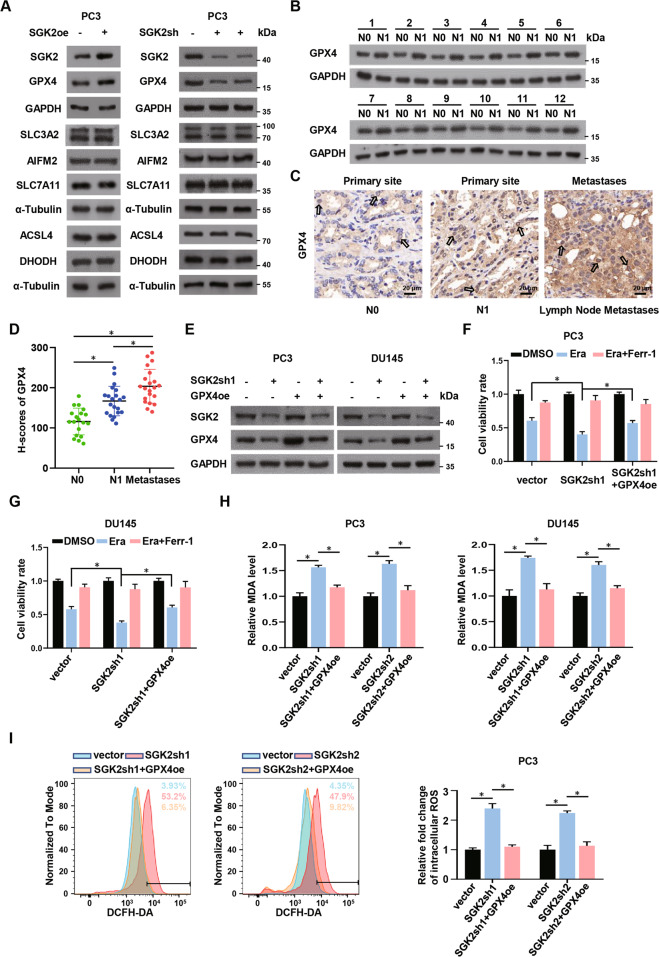
SGK2 knockdown facilitates ferroptosis by downregulating GPX4. **A** Protein levels of ferroptosis-related genes were examined in SGK2 overexpressed or knockdown PC3 cells by western blotting. **B** Western blotting analysis of GPX4 expression in PCa tissues without lymph node metastasis(N0) and PCa tissues with lymph node metastasis(N1). **C**, **D** Immunohistochemistry (IHC) analysis of GPX4 expression difference and H-scores of GPX4 among PCa tissues without lymph node metastasis(N0) and PCa tissues with lymph node metastasis(N1) and corresponding Lymph Node Metastases tissues. Scale bars, 20 μm. **E** Western blot assay showed the reversion efficiency of overexpressed GPX4 after SGK2 knockdown in PCa. **F**, **G** Cell viability of PCa cells with SGK2 knockdown, with/without GPX4 overexpression, were measured after treating with Era (5 μM), Era (5 μM) and Ferr-1(5 μM) for 24 h by CCK-8 assay. **H** Concentrations of MDA were measured in the indicated group. **I** Intracellular ROS of the indicated group was stained by DCFH-DA and determined by flow cytometry. Data are presented as representative images or as the mean ± SD of three independent experiments. *P < 0.05.

To further explore the mechanism by which SGK2 downregulation promoting ferroptosis, we investigated ferroptosis-related proteins that may be affected by both SGK2 downregulation and upregulation in PCa cell lines. Consistent with SGK2 expression level, western blotting analysis demonstrated that GPX4 levels were markedly reduced in PC3 and DU145 cells when SGK2 was stably knocked down. However, the expression of other ferroptosis-related proteins (SLC3A2, AIFM2, SLC7A11, ACSL4, and DHODH) [26–30], did not change. SGK2 overexpression increased GPX4 levels, but not that of other ferroptosis-related proteins (Fig. 3A; Supplementary Fig. S6A, B). Besides, the qRT-PCR assays showed that SGK2 overexpression increased GPX4 mRNA levels, while SGK2 knockdown decreased GPX4 mRNA levels (Supplementary Fig. S6C, D). Moreover, GPX4 protein levels were higher in PCa tissues with LNM than in PCa tissues without LNM (Fig. 3B; Supplementary Fig. S6E). In addition, we found that GPX4 expression levels gradually increased in PCa without LNM, PCa with LNM, and corresponding lymph node metastases using immunohistochemical analysis (Fig. 3C, D). This finding was consistent with SGK2 expression levels in PCa tissues. Based on these data, we speculated that GPX4 may be an important downstream molecule of SGK2 downregulation facilitating ferroptosis.

To confirm this hypothesis, we conducted rescue experiments using PC3 and DU145 cells. First, western blotting showed that GPX4 overexpression reversed the decline in GPX4 protein levels caused by SGK2 downregulation (Fig. 3E; Supplementary Fig. S6F). In the CCK-8 assay, we observed that GPX4 overexpression promoted cancer cell growth, which was inhibited by low levels of SGK2 (Fig. 3F, G). In addition, GPX4 overexpression inhibited lipid peroxidation (which was triggered by SGK2 downregulation) in SGK2-silenced PC3 and DU145 cells (Fig. 3H, I; Supplementary Fig. S6G). Taken together, our results indicated that SGK2 reduction contributed to ferroptosis by repressing GPX4 expression.

### GPX4 rescues the progression of SGK2 knockdown in PCa cells

**Fig. 4:**
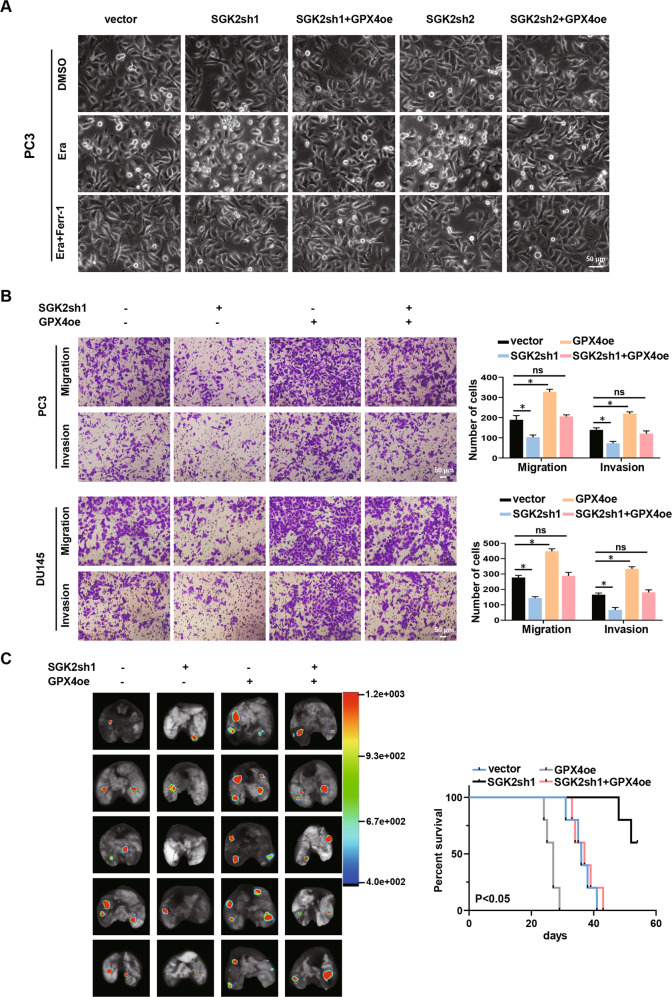
GPX4 rescues the progression of SGK2 knockdown in PCa cells. **A** Representative phase-contrast images of PC3 cells with SGK2 knockdown, with/without GPX4 overexpression after treating with Era (5 μM), Era (5 μM) and Ferr-1(5 μM) for 24 h. Scale bars, 50 µm. **B** Transwell analysis of the migration and invasion capability of PCa cells stably transfected as indicated. Scale bars, 50 μm. **C** Left panel, representative bioluminescent image of metastatic lung colonization in nude mice injected with corresponding cells via tail vein. Right panel, Kaplan–Meier curves for nude mice in the metastasis assay. Data are presented as representative images or as the mean ± SD of three independent experiments. *P < 0.05; ns, not significant.

In vitro and in vivo experiments were conducted to verify whether SGK2 exerts its biological effects through GPX4. Using an inverted phase-contrast microscope, we observed that GPX4 upregulation restored the shrinkage and deterioration of cell state caused by SGK2 downregulation (Fig. 4A; Supplementary Fig. S7A). Transwell experiments showed that GPX4 overexpression increased the migration and invasion abilities of PC3 and DU145 cells. SGK2 knockdown reduces metastatic capacity, but GPX4 overexpression reversed this effect, thereby promoting metastatic capacity (Fig. 4B). We established a model of lung metastasis in athymic nude mice by injecting red fluorescent PC3 cells into the tail vein. Consistent with the above in vitro results, we found that SGK2 knockdown significantly decreased the number of pulmonary metastases as seen in bioluminescence images and observed that mice had a greater probability of survival. However, GPX4 overexpression significantly recused the decrease of pulmonary metastases and the increase of survival probability caused by SGK2 knockdown. (Fig. 4C). These results confirmed that SGK2 downregulation inhibited PCa progression by downregulating GPX4 expression in vitro and in vivo.

### SGK2 downregulates GPX4 expression by promoting the nuclear exclusion of FOXO1

**Fig. 5:**
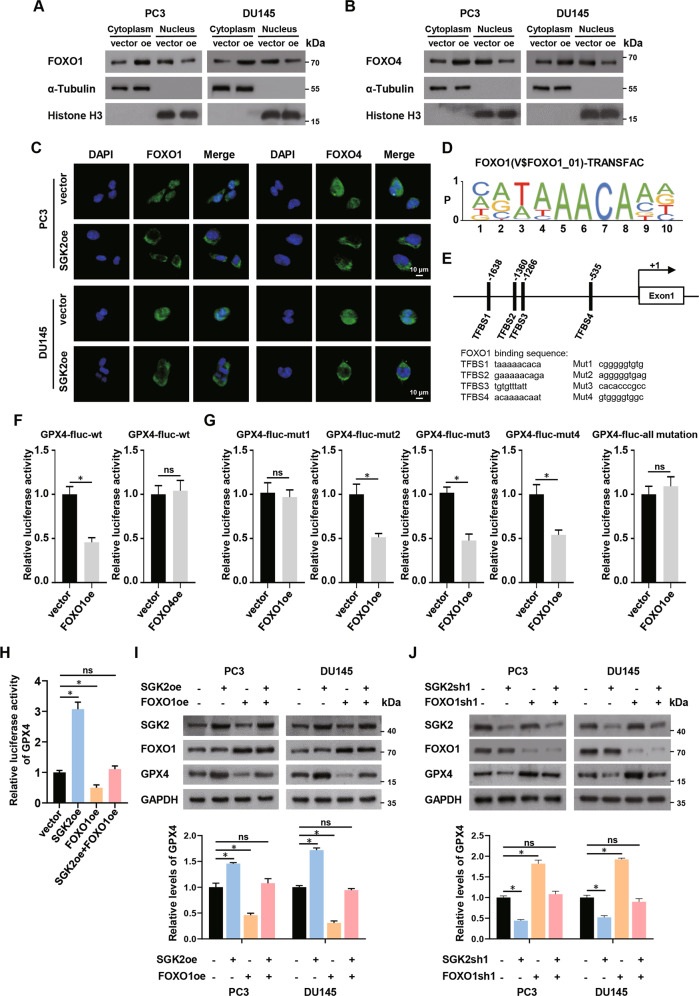
SGK2 downregulates GPX4 expression by promoting the nuclear exclusion of FOXO1. **A**, **B** Levels of cytoplasmic and nuclear FOXO1/FOXO4 protein in PCa cells with SGK2 overexpression were determined by western blotting. α-Tubulin and Histone-H3 were used as cytoplasmic and nuclear markers, respectively. **C** Location of FOXO1/FOXO4 in PCa cells with SGK2 overexpression was detected by immunofluorescence staining. Scale bars, 10 μm. **D** Position weight matrix of FOXO1 binding site motif from TRANSFAC database. **E** Pattern of four predicted transcription factor binding sites of FOXO1 and GPX4 promoter by using the ConTra V2 database, and corresponding mutant sites. (TFBS, transcription factor binding sites). **F** The luciferase activity of wild-type (wt) GPX4 promoter after transfection with FOXO1/FOXO4 overexpressed plasmids in PC3 cells. **G** The luciferase activities of the four mutant transcription factor binding sites of GPX4 promoter after transfection with FOXO1 overexpressed plasmids in PC3 cells. **H** The luciferase activity of GPX4 promoter after transfection with SGK2 overexpressed plasmids and FOXO1 overexpressed plasmids independently or in combination in PC3 cells. **I**, **J** Western blot assay showed the levels of GPX4 protein in the indicated group. Data are presented as representative images or as the mean ± SD of three independent experiments. *P < 0.05; ns, not significant.

SGK family can regulate transcription factors of the FOXO family, including FOXO1, FOXO3, FOXO4, and FOXO6 factor [31]. A previous report showed that the SGK family inhibited FOXO factor activity by promoting the transfer of FOXO factors from the nucleus to the cytoplasm [32]. However, it is unclear which members of the FOXO family are regulated by SGK2. First, we analyzed the protein expression levels of FOXO factors by separating the nuclear and cytoplasmic proteins from cells. SGK2 overexpression increased the protein levels of FOXO1 and FOXO4 in the cytoplasm, but had no significant effect on the distribution of FOXO3 and FOXO6 in the nucleus or cytoplasm (Fig. 5A, B; Supplementary Fig. S8A, B). In addition, immunofluorescence analysis showed consistent results (Fig. 5C; Supplementary Fig. S8C). Furthermore, we investigated whether FOXO1/FOXO4 could affect GPX4 expression by considering its transcriptional activity. Using the TRANSFAC and ConTra V2 databases, we predicted the binding sites of FOXO1/GPX4 and FOXO4/GPX4 (Fig. 5D, E; Supplementary Fig. S8D, E). We constructed dual luciferase reporter plasmids containing the wild-type GPX4 promoter and transfected these into PC3 cells to investigate the transcriptional functions of FOXO1 and FOXO4. Compared with the empty vector control group, FOXO1 overexpression decreased the relative reporting activity, whereas FOXO4 overexpression did not significantly affect the relative reporting activity (Fig. 5F). To further evaluate the transcriptional regulation of GPX4 by FOXO1, we designed a dual luciferase reporter plasmid containing the mutant GPX4 promoter. We found that the transcriptional inhibition of GPX4 by FOXO1 could be relieved only when transcription factor binding site 1 (TFBS1) was mutated, but mutations at other binding sites did not yield the same effects. When all the binding sites were mutated, the results were consistent with the mutation at TFBS1. Therefore, we found that FOXO1 inhibited GPX4 expression through TFBS1 (Fig. 5G). We conducted rescue experiments to determine whether SGK2 regulates GPX4 expression via FOXO1. As shown in Fig. 5H, overexpression of SGK2 elevated the relative reporting activity compared with the empty vector control group. However, FOXO1 overexpression reversed the SGK2-enhanced relative reporting activity. Western blot analysis demonstrated that FOXO1 overexpression reduced GPX4 protein levels, which was initially increased by SGK2 upregulation (Fig. 5I), and FOXO1 knockdown increased GPX4 levels, which was initially decreased by SGK2 knockdown (Fig. 5J). Based on these experimental results, we confirmed that SGK2 upregulated GPX4 expression by facilitating the nuclear exclusion of FOXO1.

### SGK2 promotes the nuclear exclusion of FOXO1 through phosphorylation of FOXO1 at Thr-24 and Ser-319

**Fig. 6:**
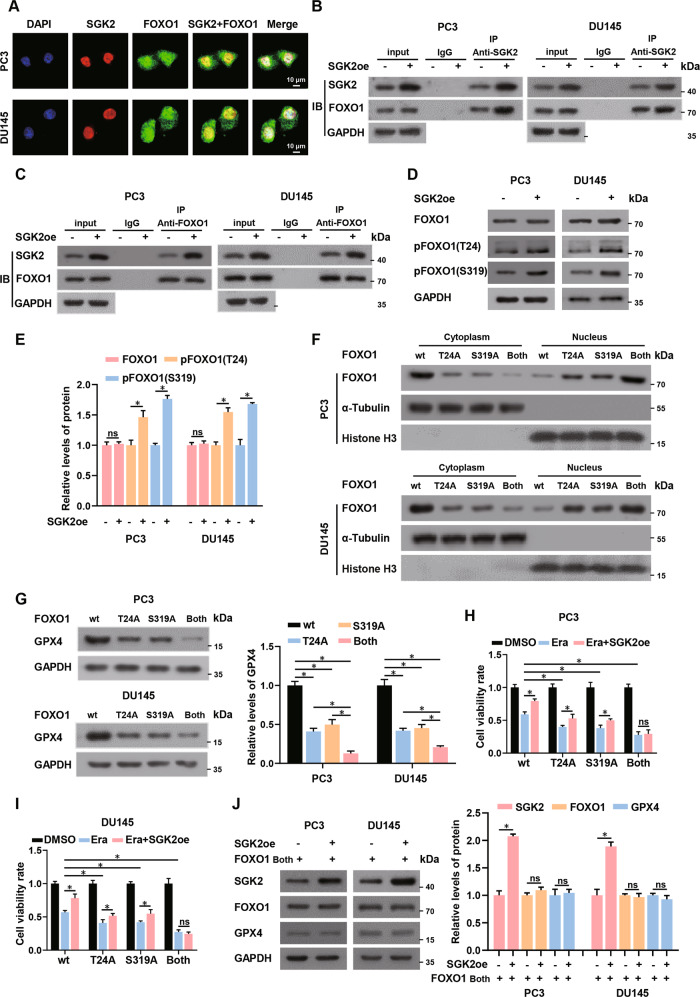
SGK2 promotes the nuclear exclusion of FOXO1 through phosphorylation of FOXO1 at Thr-24 and Ser-319. **A** Demonstrative immunofluorescence images of SGK2 and FOXO1 protein location in PC3 and DU145 cells. Scale bars, 10 μm. **B**, **C** Cross-linking of SGK2 and FOXO1 in PCa cells with SGK2 overexpression was detected by co-immunoprecipitation experiment with anti-SGK2 antibody and anti-FOXO1 antibody. The lysate immunoprecipitated with anti-immunoglobulin G antibody was served as negative control. **D**, **E** Western blotting analysis of the expression level of FOXO1 phosphorylation at Thr-24(T24) and Ser-319(S319) in PCa cells stably overexpressing SGK2. **F** On the basis of SGK2 overexpression, levels of cytoplasmic and nuclear FOXO1 protein in the indicated group by western blotting. α-Tubulin and Histone-H3 were used as cytoplasmic and nuclear markers, respectively. **G** On the basis of SGK2 overexpression, western blotting analysis of the expression level of GPX4 in PCa cells transfected with FOXO1 overexpressed plasmids of wt, T24A, S319A, Both, respectively. **H**, **I** Cell viability was detected by CCK-8 assay in the indicated group. **J** FOXO1 and GPX4 expression levels were assessed using western blotting in PCa cells with/without SGK2 overexpression. **F**–**J** wt, wild-type FOXO1 overexpressed plasmids; T24A, mutant T24A FOXO1 overexpressed plasmids; S319A, mutant S319A FOXO1 overexpressed plasmids; Both, mutant T24A and S319A FOXO1 overexpressed plasmids. Data are presented as representative images or as the mean ± SD of three independent experiments. *P < 0.05; ns, not significant.

It was previously reported that FOXO1 could be phosphorylated at Ser-256 (S256), Thr-24 (T24) and Ser-319 (S319) and that its nuclear exclusion could be thus promoted [33]. We assessed whether SGK2 overexpression facilitated the nuclear exclusion of FOXO1 by phosphorylation at the specific FOXO1 sites in PCa cells. Immunofluorescence assays revealed significant co-localization of SGK2 and FOXO1 in PC3 and DU145 cells (Fig. 6A). In addition, co-immunoprecipitation experiments detected cross- linked SGK2 and FOXO1 in PC3 and DU145 cells, showing obvious co-localization (Fig. 6B, C). Western blotting results showed that SGK2 overexpression significantly enhanced phosphorylation levels of FOXO1 at T24 and S319, instead of S256; however, SGK2 overexpression did not affect the total FOXO1 protein levels and mRNA levels compared with the control group (Fig. 6D, E; Supplementary Fig. S9A, B). Mutations of T24 and S319 amino acid residues to alanine led to phosphorylation loss at both sites thereby inhibiting the translocation of FOXO1 [33]. To further verify whether SGK2 phosphorylates T24 and S319 of FOXO1 in PCa and promotes the nuclear exclusion of FOXO1, we established a stable knockdown of FOXO1 in PC3 and DU145 cell lines, and transfected plasmids expressing threonine-alanine (T24A) and/or serine-alanine (S319A) mutant FOXO1 (Supplementary Fig. S9C). Furthermore, we transfected SGK2 overexpression plasmids to better study the effect of SGK2 on FOXO1 phosphorylation. Proteins in the cells were separated into cytosolic and nuclear fractions. When only one site (T24A or S319A) was mutated, translocation of FOXO1 from the nucleus to the cytoplasm was inhibited to a slight extent, compared with the control group. When both sites were mutated, FOXO1 nuclear translocation was significantly inhibited, and FOXO1 was mainly distributed in the nucleus at that time (Fig. 6F). When SGK2 was overexpressed, T24A or S319A moderately inhibited GPX4 expression, compared with wild-type FOXO1, whereas the combined mutation markedly suppressed GPX4 expression (Fig. 6G). We observed consistent results in the CCK-8 assay; however, overexpression of SGK2 partially reversed the decrease in cell survival caused by T24A or S319A, but had almost no significant effect on cell survival influenced by the combined mutation (Fig. 6H, I). In addition, western blotting showed that SGK2 overexpression failed to regulate GPX4 expression when both sites were mutated, compared with that in the control group (Fig. 6J). Besides, overexpression of SGK2 did not affect MDA and ROS levels when both sites were mutated (Supplementary Fig. S9D, E). Overall, these results confirmed that SGK2 overexpression facilitated the nuclear exclusion of FOXO1 through phosphorylation at T24 and S319, thereby indirectly upregulating GPX4 expression.

## Discussion

Positive lymph nodes after radical prostatectomy are an adverse pathological feature that usually indicates a poor prognosis for PCa patients [34]. Metastatic cells can evade programmed cell death, including apoptosis, autophagic cell death, necroptosis, and ferroptosis [35, 36]. Ferroptosis is an excessive lipid peroxidation-induced cell death that can suppress tumor invasion. Ferroptosis plays a critical role in the metastasis of melanoma and mesothelioma [25, 37]. Our previous study revealed that resistance to ferroptosis promoted PCa metastasis [38]. In this study, we focused on the mechanisms by which PCa cells escape ferroptosis and systematically identified the SGK2 gene. The increase of SGK2 reportedly promoted cancer progression [39, 40]. However, it remains unclear whether SGK2 affects PCa progression. In this study, we found that SGK2 expression levels were higher in metastatic PCa samples from our center, and the results were consistent with samples from TCGA and GEO databases. In addition, an increase in SGK2 expression was strongly correlated with poor OS and PFS in TCGA database. SGK2 overexpression significantly enhanced migration and invasion abilities, whereas SGK2 knockdown weakened metastatic capacity. Based on these data, we proposed that the increase of SGK2 played an important role in promoting PCa progression.

ROS plays an important role in ferroptosis [41]. The inhibition of SLC7A11, GPX4, P53, etc. causes ROS accumulation, which promotes oxidative cell death and eventually leads to ferroptosis [42–44]. Ferroptosis caused by ROS accumulation has been demonstrated in tumors, neurological diseases, and trauma [13, 45, 46]. Currently, many studies have confirmed that the progression of PCa is related to ROS accumulation. PMANs inhibited the expression of GPX4 and SLC7A11, which enhanced ROS accumulation and caused ferroptosis in PCa [47]. In addition, our previous study also showed that CEMIP promoted the survival of detachment-resistant PCa cells by reducing ROS levels and inhibiting ferroptosis via enhancing SLC7A11 expression [38]. In the study, SGK2 was related to oxidative stress [8], and GSEA enrichment analysis showed that SGK2 was correlated with oxidative phosphorylation and the ROS pathway. Therefore, we hypothesized that SGK2 upregulation in facilitating PCa metastasis through a ferroptosis mechanism. Recent studies have also reported that inhibiting ferroptosis promotes PCa progression [48, 49]. In our study, SGK2 upregulation decreased ROS and MDA levels and inhibited erastin-induced cell death. By detecting several major ferroptosis-related proteins, we found that GPX4 levels were regulated by SGK2. We speculated that SGK2 may promote PCa metastasis by increasing the expression of GPX4. GPX4 is a major ferroptosis regulator that uses glutathione to protect cells from ferroptosis by eliminating phospholipid peroxides [50]. In vivo and in vitro studies, we found that GPX4 overexpression reversed the decrease in metastatic ability caused by SGK2 knockdown, suggesting that SGK2 promoted PCa metastasis by upregulating GPX4 expression. Moreover, this finding was consistent with the results of other studies. Liu et al. found that dysregulated cholesterol homeostasis resulted in resistance to ferroptosis and increased tumorigenicity and metastasis in breast cancer [14]. In addition, Zou et al. showed that GPX4 greatly sensitized clear cell renal cell carcinoma and ovarian cancer cells to ferroptosis [51]. In this study, we revealed a novel role for SGK2 in ferroptosis regulation in PCa.

The SGK family alters the subcellular localization of the transcription factor FOXO by phosphorylating FOXO factors [31, 32]. FOXO factors regulate the expression of genes related to oxidative stress, glucose metabolism, DNA damage repair, apoptosis, and cell differentiation [31, 32]. FOXO proteins act as tumor suppressors and are inactivated in many human cancers [52, 53]. In our study, we observed that SGK2 overexpression increased the nuclear exclusion of FOXO1. Using a dual- luciferase reporter assay, we found that FOXO1 could negatively regulate GPX4. FOXO1 overexpression reversed the increase of GPX4 relative reporter activity and protein levels induced by SGK2 overexpression; therefore, we further confirmed that SGK2 upregulated GPX4 expression through FOXO1. S256, T24, and S319 are phosphorylation sites of the FOXO1 protein, and it was reported that S256, T24, and S319 could be phosphorylated by PKB to promote the nuclear exclusion of FOXO1 [33]. PKB and SGK2 both belong to AGC kinase family [54]. However, it remains unclear whether FOXO1 could be phosphorylated at S256, T24 or S319 by SGK2. In our study, western blotting results revealed that SGK2 overexpression brought no significant changes in S256 phosphorylation level of FOXO1, in contrast to T24 and S319, of which phosphorylation levels were both significantly elevated in PCa cells. Hence, T24 and S319 were further selected in site mutation study. The residues of T24 and S319 were mutated into alanine, which prevented phosphorylation at the corresponding site in FOXO1 [33]. Single and combined mutations of T24A and S319A blocked the nuclear exclusion of FOXO1 to various extents, correspondingly reducing the expression of GPX4 to varying degrees. Interestingly, when both T24A and S319A were mutated, SGK2 overexpression almost completely lost its role in upregulating GPX4.

This study has several limitations. First, the collected clinical samples were inadequate to promote the credibility of this study, and we will continue to collect clinical samples of PCa in the future. Second, in our study, we observed that SGK2 overexpression promoted the nuclear exclusion of FOXO1 by increasing the phosphorylation level of FOXO1. However, we did not explore the specific mechanism of FOXO1 nuclear exclusion. Ng et al. reported that T24 phosphorylation triggers 14-3-3 protein binding and export of FOXO1 to the cytoplasm [55]. It is unclear how S319 phosphorylation affects the nuclear exclusion of FOXO1. Future studies should continue to explore these relevant mechanisms.

**Fig. 7:**
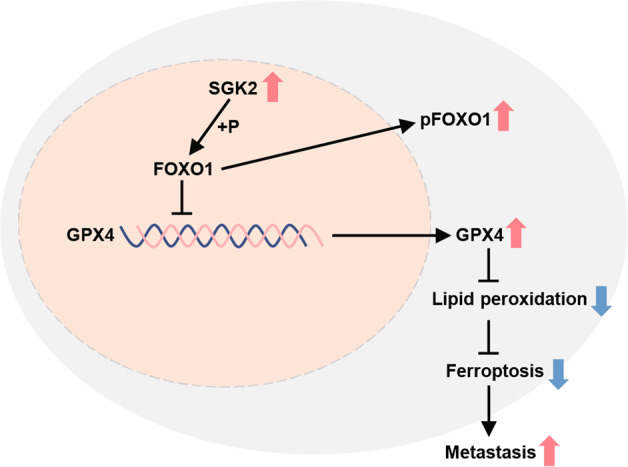
Schematic illustration of promoting metastasis in PCa cells by inhibition of ferroptosis through SGK2/FOXO1/GPX4 axis. The upregulation of SGK2 increased the nuclear exclusion of FOXO1 via phosphorylating FOXO1 and indirectly upregulated GPX4 expression, which ultimately suppressed ferroptosis and promoted PCa metastasis.

In conclusion, we demonstrated that the upregulation of SGK2 suppressed ferroptosis by increasing the nuclear exclusion of FOXO1 via phosphorylation at specific FOXO1 sites and indirectly upregulating GPX4 expression, ultimately promoting PCa metastasis (Fig. 7). These findings may translate into new therapies for the prevention and treatment of metastatic PCa.

## Supplementary information

Supplementary Figure and Table legends

Supplementary Figure S1

Supplementary Figure S2

Supplementary Figure S3

Supplementary Figure S4

Supplementary Figure S5

Supplementary Figure S6

Supplementary Figure S7

Supplementary Figure S8

Supplementary Figure S9

Supplementary Table S1

Supplementary Table S2

Supplementary Table S3

Supplementary Table S4

Supplementary Table S5

Original western blots

Reproducibility Checklist

## Data Availability

The data used and/or analyzed during the study are available from the corresponding author on reasonable request.
