# Supplementary material for: SGK2 promotes prostate cancer metastasis by inhibiting ferroptosis via upregulating GPX4
Source: Cell Death Dis. 2023 Jan 31;14(1):74. doi: 10.1038/s41419-023-05614-5 (PMC9889330; doi:10.1038/s41419-023-05614-5)
Supplement: Supplementary file 1 — Supplementary Figure and Table legends [file 41419_2023_5614_MOESM1_ESM.docx]

**Supplementary Figure and Table legends**

**Supplementary Figure S1. The survival and prognosis analysis of upregulated candidate genes**

**(A)** Overall survival and progression free survival were analyzed according to high or low expression of upregulated candidate genes via the TCGA datasets. The number of patients in low expression groups is 249, and the number of patients in high expression groups is 250. (The gene expression level of TM4SF5 was the minimum (0) in more than half of the samples in the TCGA datasets, which could not be divided into high and low expression groups, resulting in failure of survival and prognosis analysis.)

**Supplementary Figure S2. The survival and prognosis analysis of downregulated candidate genes**

**(A)** Overall survival and progression free survival were analyzed according to high or low expression of downregulated candidate genes via the TCGA datasets. The number of patients in low expression groups is 249, and the number of patients in high expression groups is 250.

**Supplementary Figure S3. SGK2 overexpression promoted the migration and invasion capability of** **PCa cells**

**(A)** Western blotting analysis of overexpression and knockdown efficiency of SGK2 in PC3 and DU145 cells.

**(B)** Transwell analysis of the migration and invasion capability of PCa cells with overexpression of SGK2. Scale bars, 50 μm.

Data are presented as representative images or as the mean ± SD of three independent experiments. *P < 0.05

**Supplementary Figure S4. SGK2 is a key negative regulator of ferroptosis in PCa**

**(A)** Representative phase-contrast images of DU145 cells with SGK2 knockdown treated with Era (5 μM), Era (5 μM) combined with Ferr-1(5 μM), Z-VAD (20 μM), or 3-MA (10 mM) for 24h, respectively. Scale bars, 50 µm.

**(B)** Intracellular ROS of DU145 cells with SGK2 knockdown was stained by DCFH-DA and determined by flow cytometry.

**(C-D)** Bar graphs showing cell viability in SGK2 overexpressed PCa cells treated with Era (5 μM), Era (5 μM) combined with Ferr-1(5 μM), Z-VAD (20 μM), or 3-MA (10 mM) for 24h, respectively.

**(E)** Intracellular ROS of PCa cells with SGK2 overexpression was stained by DCFH-DA and determined by flow cytometry.

**(F)** Concentrations of MDA were measured in PCa cells with SGK2 overexpression.

**(G)** The morphological changes of mitochondria were detected by TEM in DU145 cells with SGK2 knockdown. Scale bars represent 2.5 µm and 1 µm, respectively.

Data are presented as representative images or as the mean ± SD of three independent experiments. *P < 0.05

**Supplementary Figure S5. Detection of mitochondrial membrane potential and intracellular Fe^2+^ in PC3 and DU145 cells**

**(A-B)** The mitochondrial membrane potential levels of PC3 and DU145 cells were detected by JC-1 staining by flow cytometry.

**(C-D)** Intracellular Fe^2+^ levels of PC3 and DU145 cells were detected by FeRhoNox™-1 fluorescent probe by confocal microscope. Scale bars, 10 μm.

Data are presented as representative images or as the mean ± SD of three independent experiments. *P < 0.05

**Supplementary Figure S6. SGK2 knockdown facilitates ferroptosis by downregulating GPX4**

**(A)** Protein levels of ferroptosis-related genes were examined in SGK2 overexpressed or knockdown PC3 cells by western blotting.

**(B)** Protein levels of ferroptosis-related genes were examined in SGK2 overexpressed or knockdown DU145 cells by western blotting.

**(C-D)** Relative mRNA levels of SGK2 and GPX4 in the indicated group were detected by qRT-PCR.

**(E)** Western blotting analysis of GPX4 expression in PCa tissues without lymph node metastasis(N0) and PCa tissues with lymph node metastasis(N1).

**(F)** Western blot assay showed the reversion efficiency of overexpressed GPX4 after SGK2 knockdown in PCa.

**(G)** Intracellular ROS of the indicated group was stained by DCFH-DA and determined by flow cytometry.

Data are presented as representative images or as the mean ± SD of three independent experiments. *P < 0.05; ns, not significant

**Supplementary Figure S7. GPX4 rescues the progression of SGK2 knockdown in DU145 cells**

**(A)** Representative phase-contrast images of DU145 cells with SGK2 knockdown, with/without GPX4 overexpression after treating with Era (5 μM), Era (5 μM) and Ferr-1(5 μM) for 24h. Scale bars, 50 µm.

Data are presented as representative images of three independent experiments.

**Supplementary Figure S8. SGK2 do not regulate nuclear translocation of FOXO3 and FOXO6**

**(A-B)** Levels of cytoplasmic and nuclear FOXO3/FOXO6 protein in PCa cells with SGK2 overexpression were determined by western blotting. α-Tubulin and Histone H3 were used as cytoplasmic and nuclear markers, respectively.

**(C)** Location of FOXO3/FOXO6 in PCa cells with SGK2 overexpression was detected by immunofluorescence staining. Scale bars, 10 μm.

**(D)** Position weight matrix of FOXO4 binding site motif from TRANSFAC database.

**(E)** Pattern of four predicted transcription factor binding sites of FOXO4 and GPX4 promoter by using the ConTra V2 database.

Data are presented as representative images of three independent experiments.

**Supplementary Figure S9. SGK2 promotes the nuclear exclusion of FOXO1 through phosphorylation of FOXO1 at Thr-24 and Ser-319**

**(A)**  Western blotting analysis of the expression level of FOXO1 phosphorylation at Ser-256(S256) in PCa cells stably overexpressing SGK2.

**(B)** Relative mRNA levels of FOXO1 in the indicated group were detected by qRT-PCR.

**(C)** Protein levels of FOXO1and FOXO1 phosphorylation at T24 and S319 were detected by western blotting in PCa cells transfected with plasmids with knocked-down FOXO1 combined with plasmids overexpressing FOXO1 of wt, T24A, S319A, Both, respectively.

**(D)** Concentrations of MDA were measured in the indicated group.

**(E)** Intracellular ROS of the indicated group was stained by DCFH-DA and determined by flow cytometry.

**(C-E)** wt, wild-type FOXO1 overexpressed plasmids; T24A, mutant T24A FOXO1 overexpressed plasmids; S319A, mutant S319A FOXO1 overexpressed plasmids; Both, mutant T24A and S319A FOXO1 overexpressed plasmids.

Data are presented as representative images of three independent experiments. *P < 0.05; ns, not significant

**Supplementary Table S1.** The sequences of primers used in the study.

**Supplementary Table S2.** Upregulated genes in PCa samples with lymph node metastasis compared with samples without lymph node metastasis from TCGA datasets (|Log_2_FC| > 1, p < 0.05).

**Supplementary Table S3.** Downregulated genes in PCa samples with lymph node metastasis compared with samples without lymph node metastasis from TCGA datasets (|Log_2_FC| > 1, p < 0.05).

**Supplementary Table S4.** Upregulated genes in PCa tissues with metastasis compared with tissues without metastasis from GEO datasets (GEO accession: GSE6752) (|Log_2_FC| > 1, p < 0.05).

**Supplementary Table S5.** Downregulated genes in PCa tissues with metastasis compared with tissues without metastasis from GEO datasets (GEO accession: GSE6752) (|Log_2_FC| > 1, p < 0.05).
