# Supplementary figures and images for: SGK2 promotes prostate cancer metastasis by inhibiting ferroptosis via upregulating GPX4
Source: Cell Death Dis. 2023 Jan 31;14(1):74. doi: 10.1038/s41419-023-05614-5 (PMC9889330; doi:10.1038/s41419-023-05614-5)

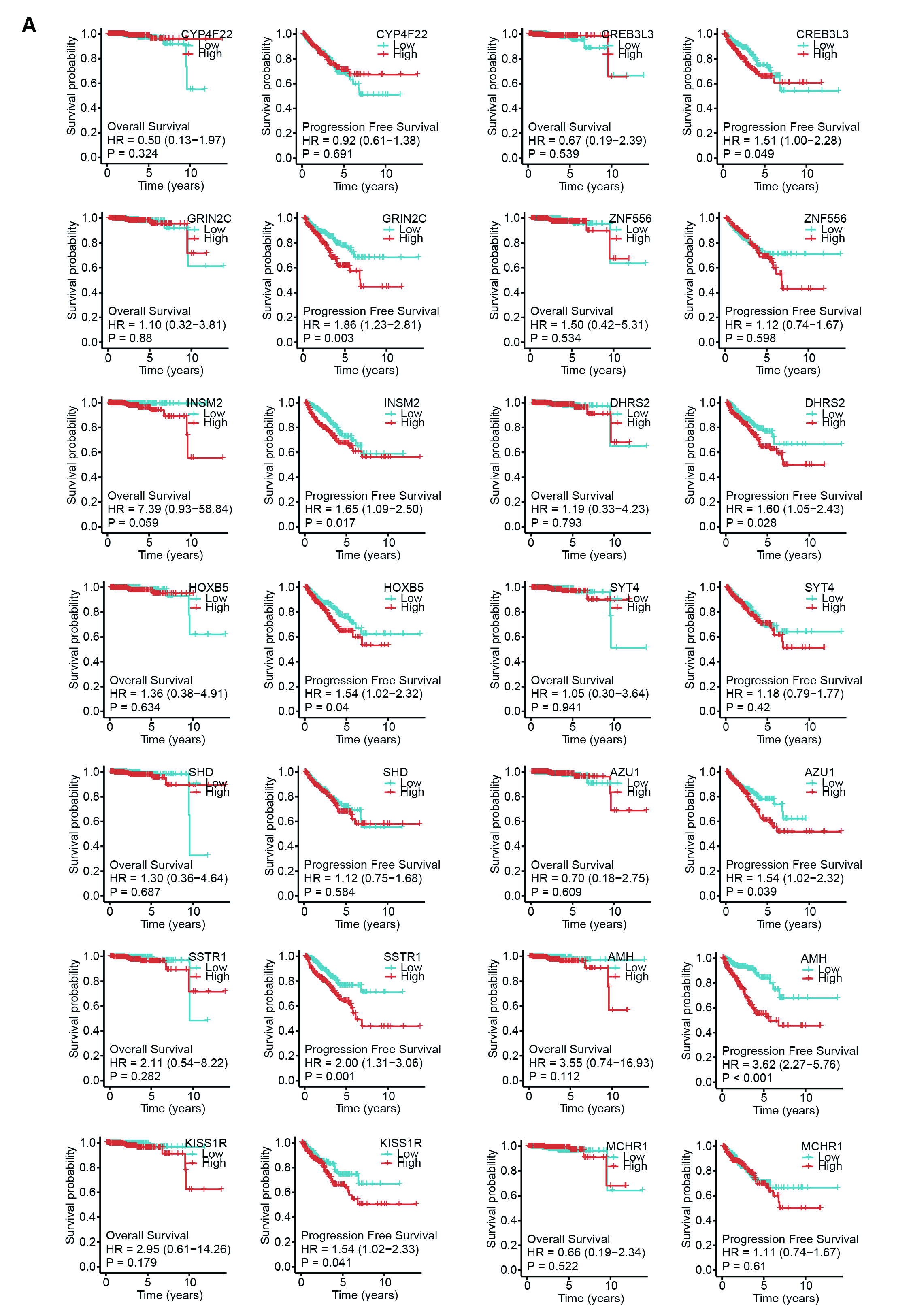

Supplement: Supplementary file 2 — Supplementary Figure S1 [file 41419_2023_5614_MOESM2_ESM.tif]

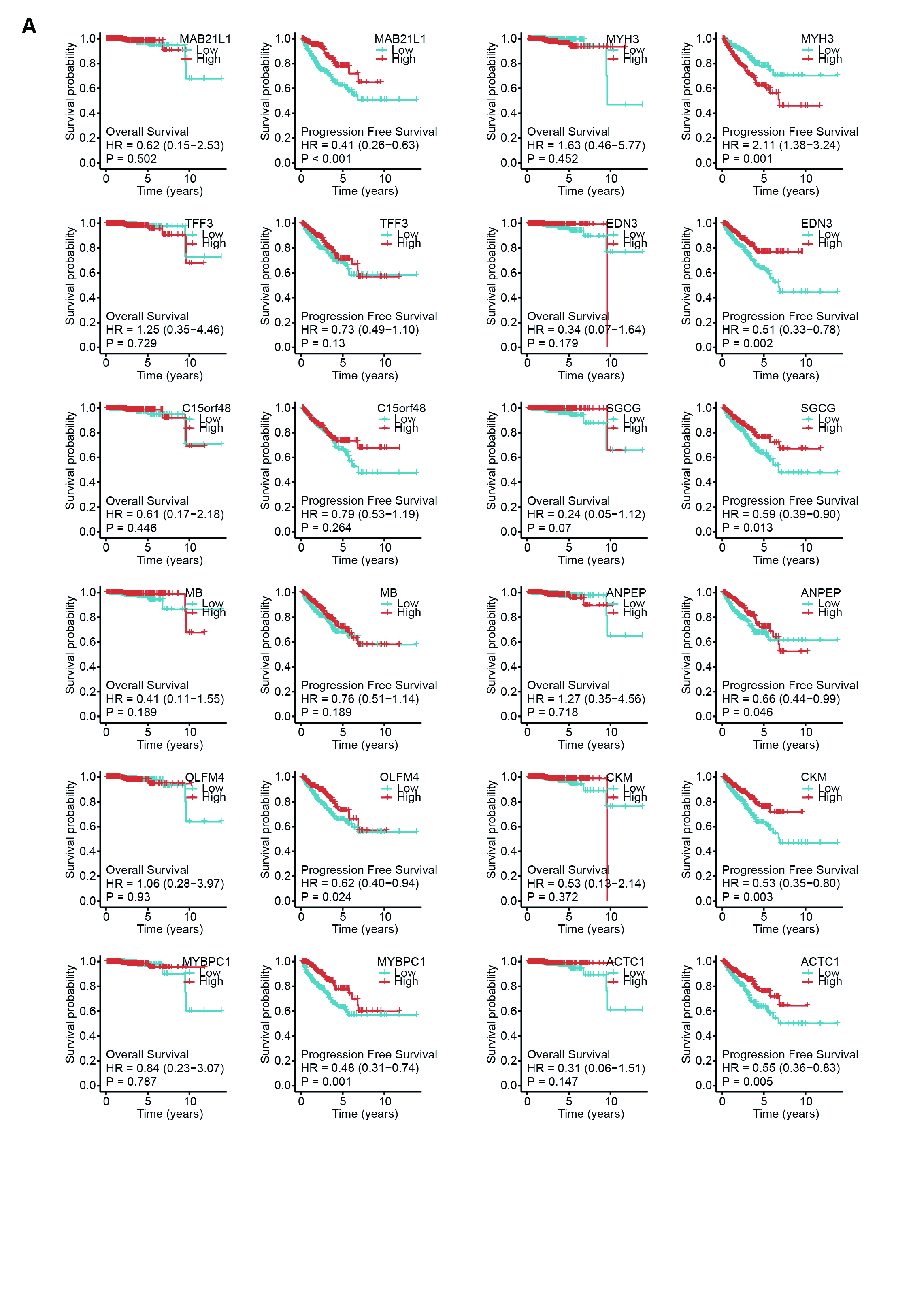

Supplement: Supplementary file 3 — Supplementary Figure S2 [file 41419_2023_5614_MOESM3_ESM.tif]

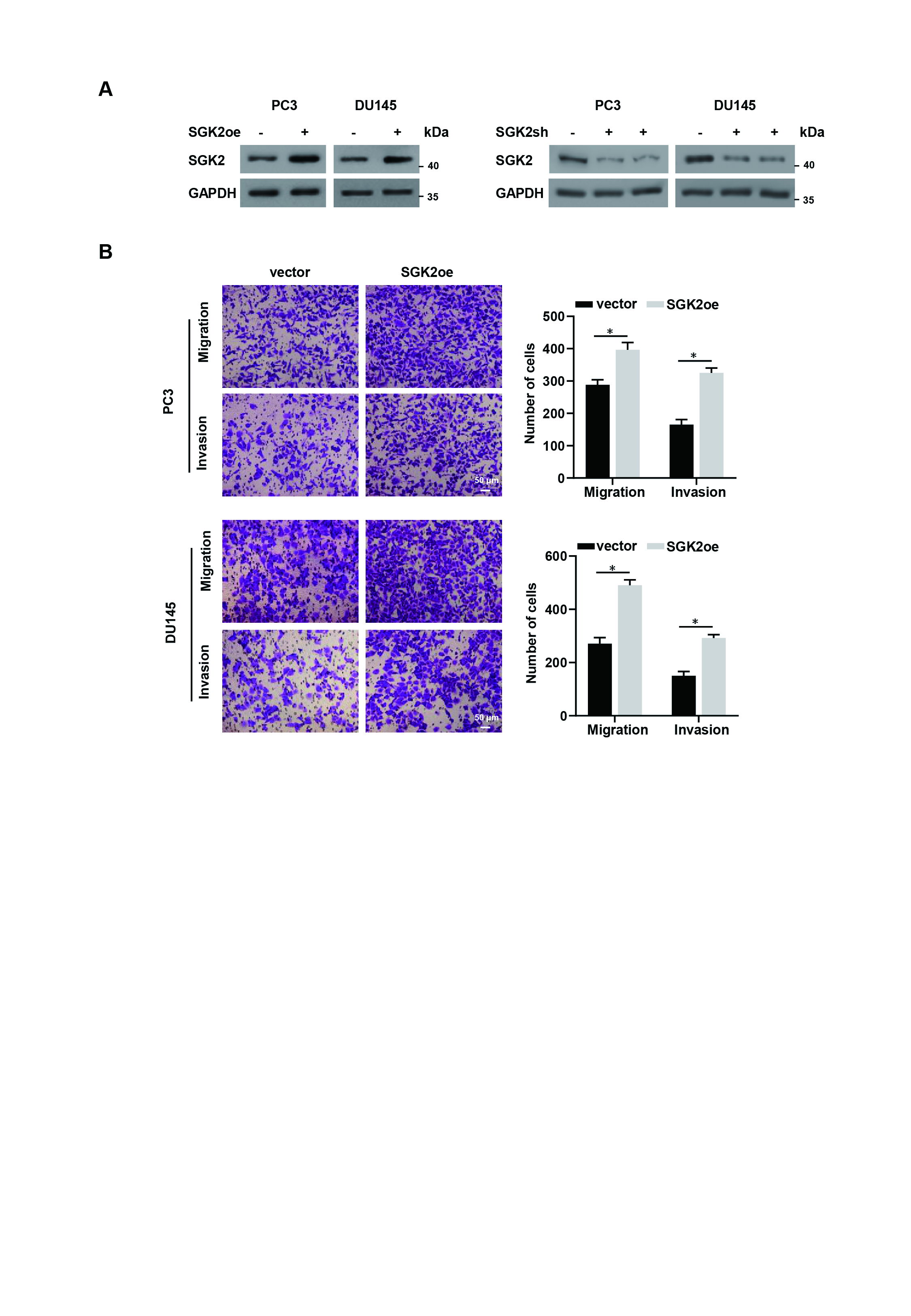

Supplement: Supplementary file 4 — Supplementary Figure S3 [file 41419_2023_5614_MOESM4_ESM.tif]

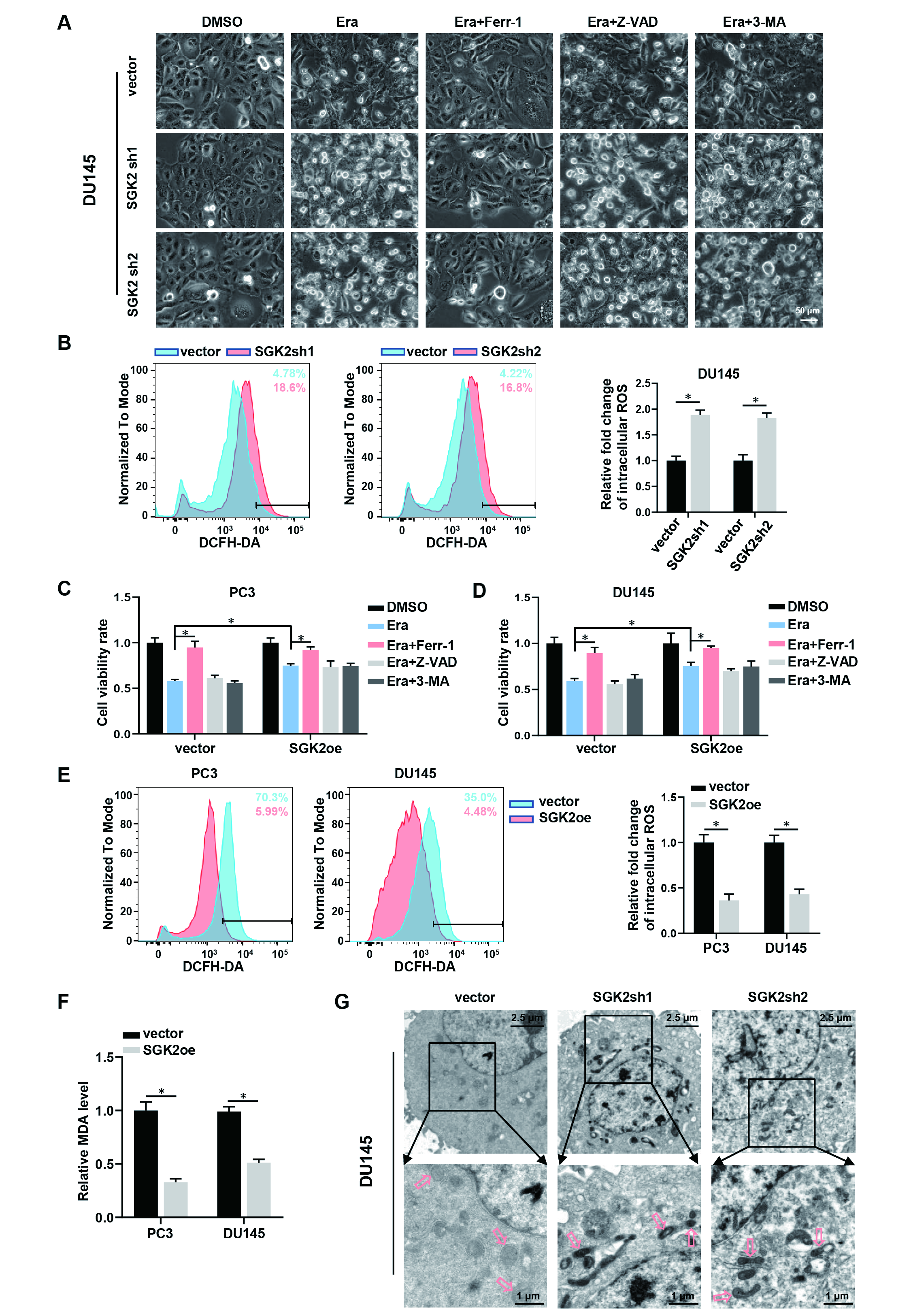

Supplement: Supplementary file 5 — Supplementary Figure S4 [file 41419_2023_5614_MOESM5_ESM.tif]

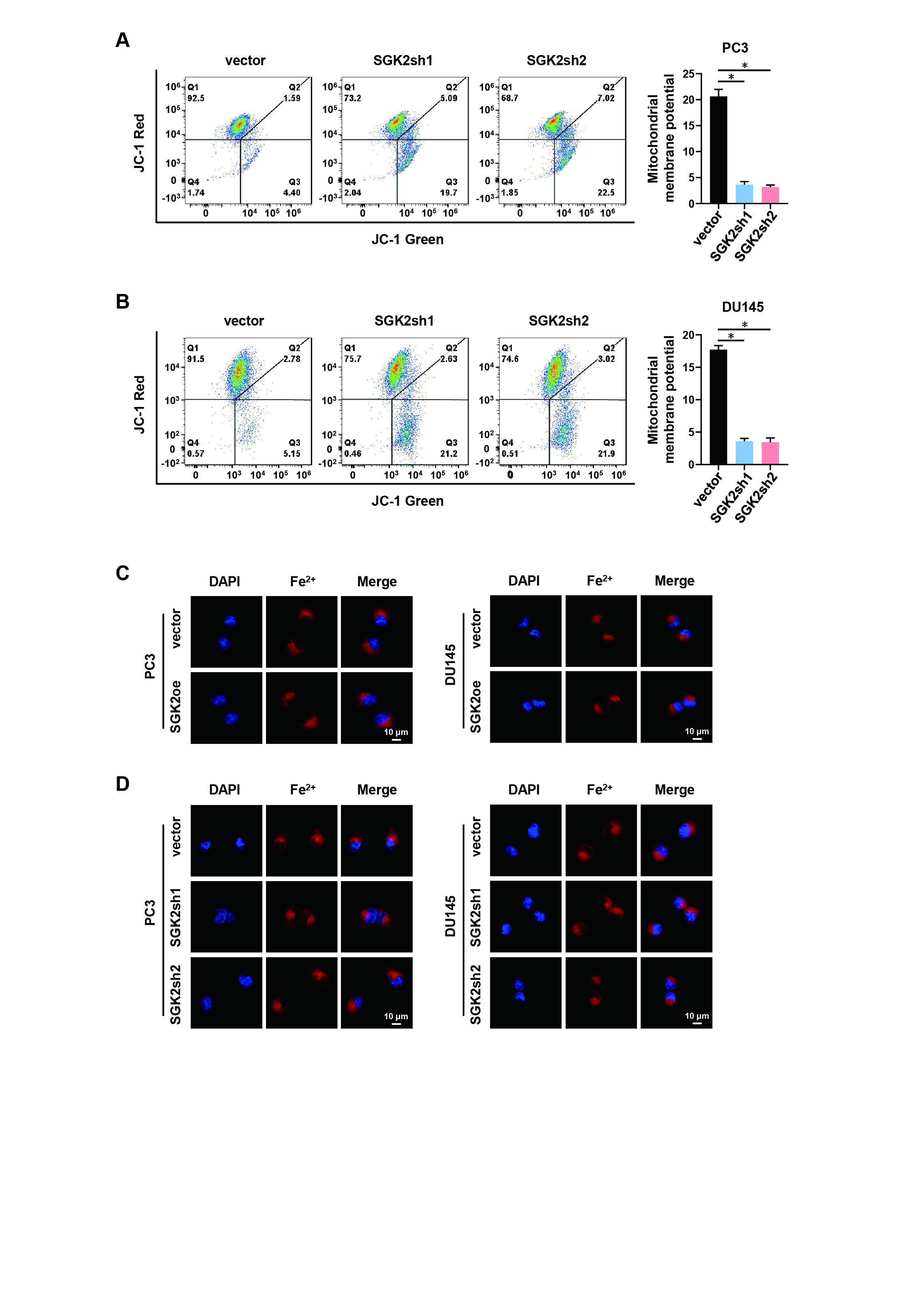

Supplement: Supplementary file 6 — Supplementary Figure S5 [file 41419_2023_5614_MOESM6_ESM.tif]

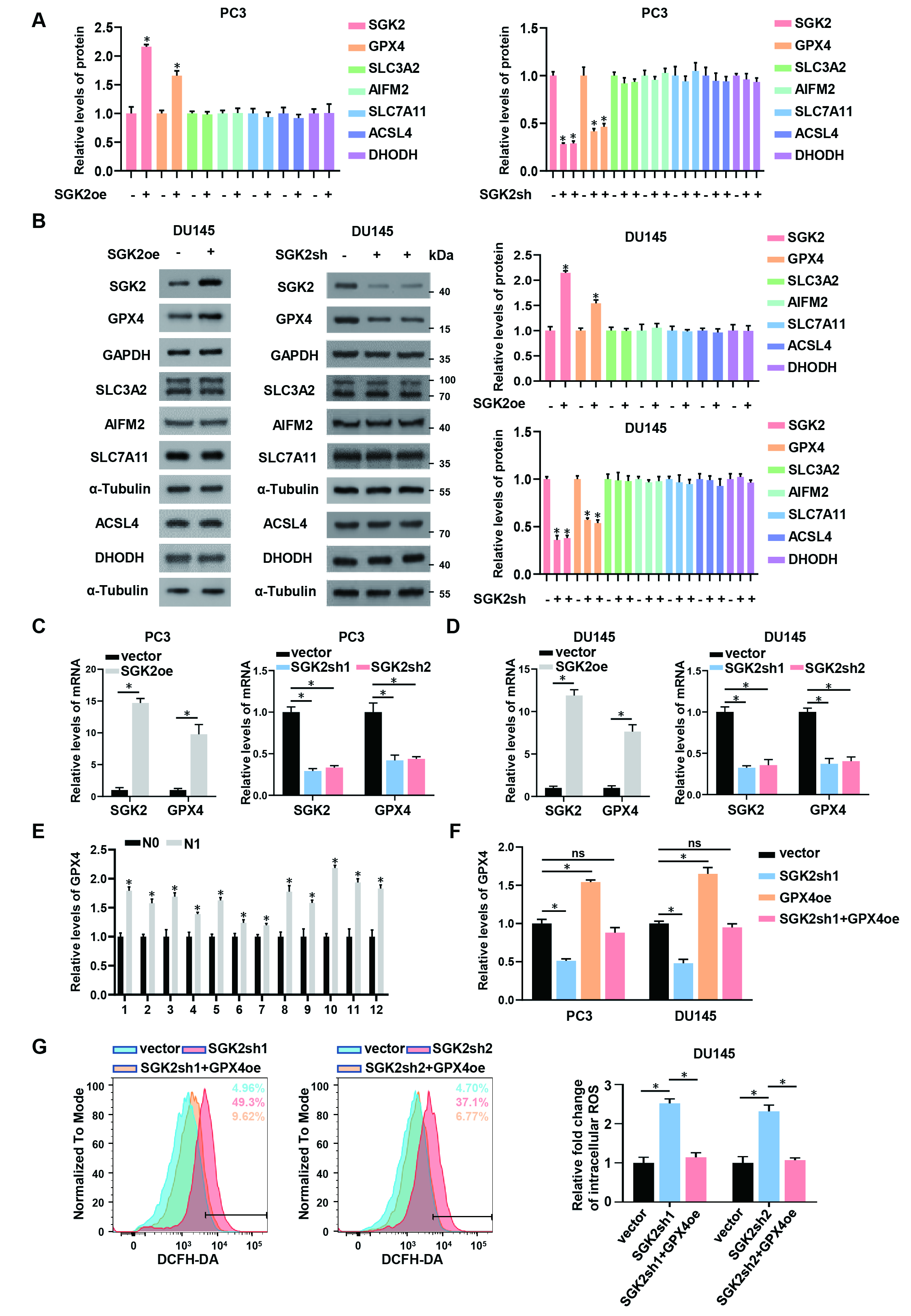

Supplement: Supplementary file 7 — Supplementary Figure S6 [file 41419_2023_5614_MOESM7_ESM.tif]

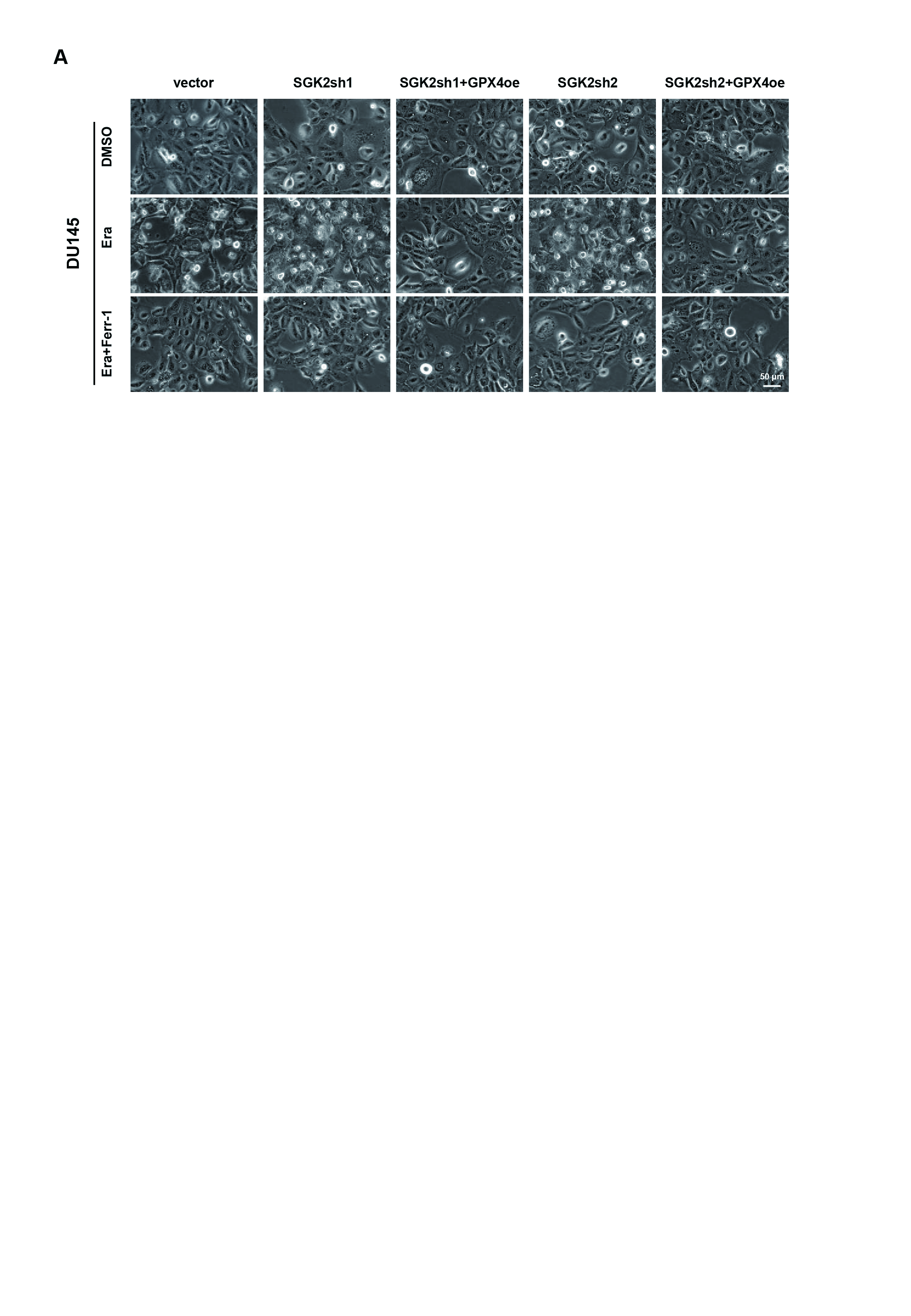

Supplement: Supplementary file 8 — Supplementary Figure S7 [file 41419_2023_5614_MOESM8_ESM.tif]

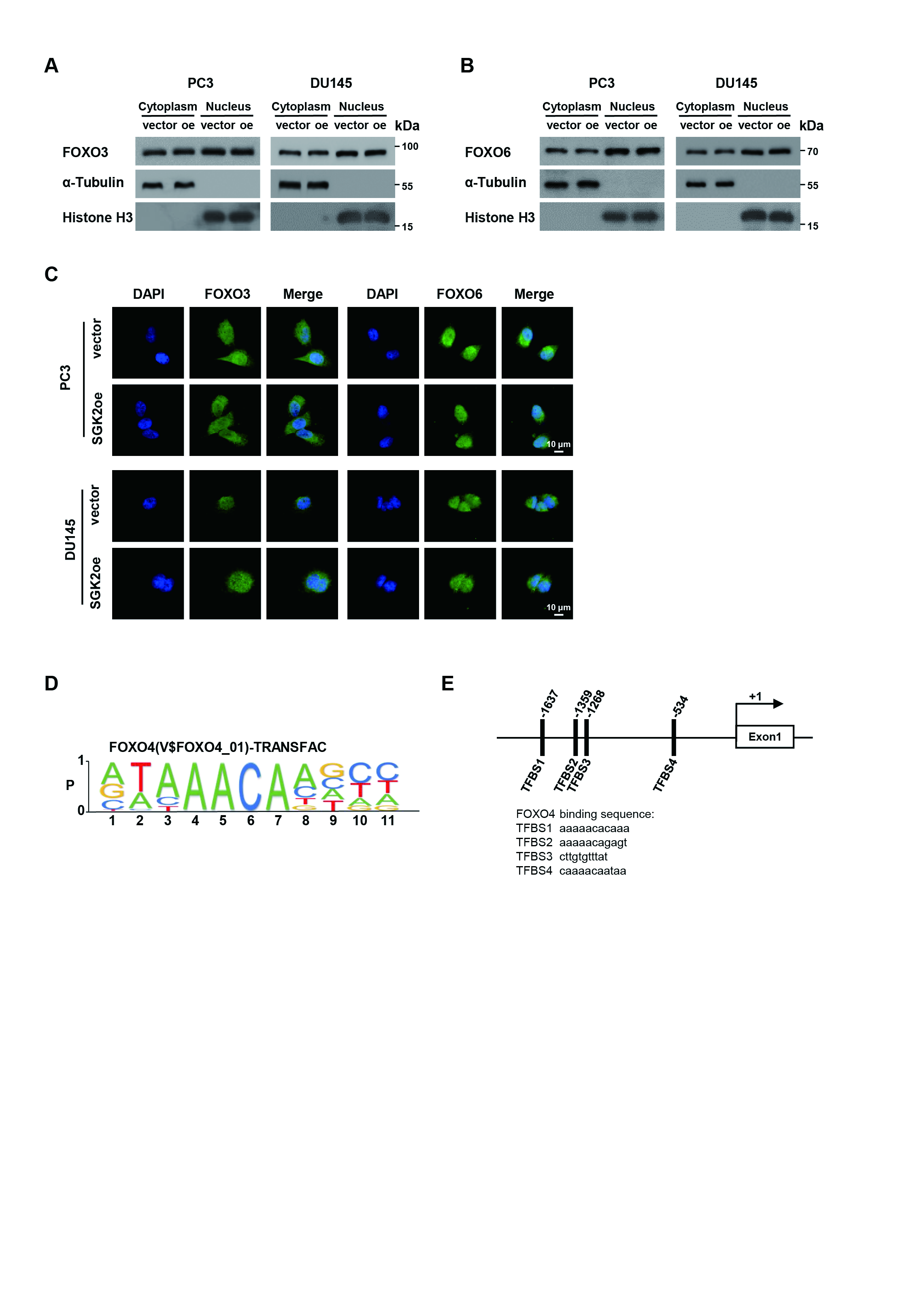

Supplement: Supplementary file 9 — Supplementary Figure S8 [file 41419_2023_5614_MOESM9_ESM.tif]

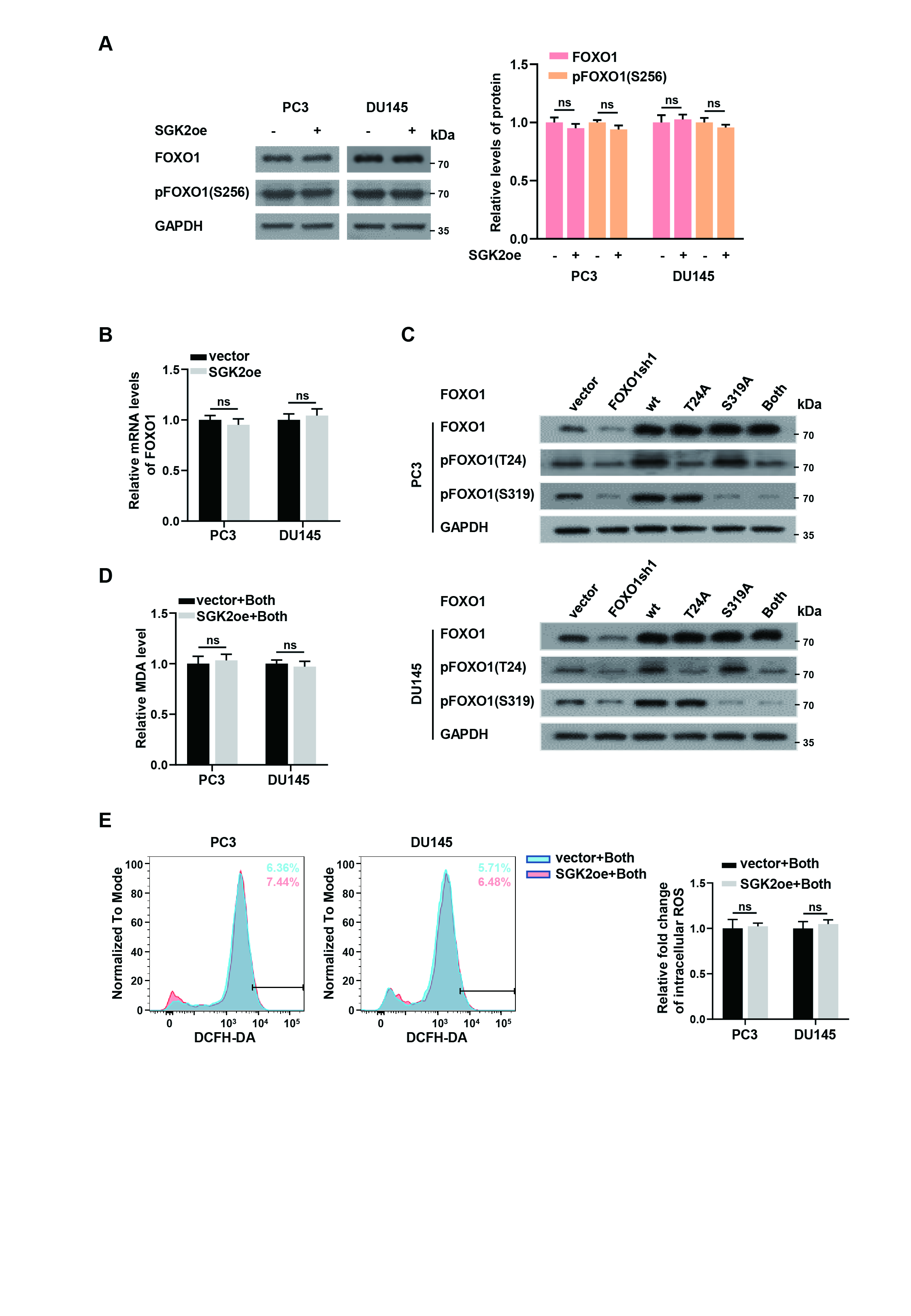

Supplement: Supplementary file 10 — Supplementary Figure S9 [file 41419_2023_5614_MOESM10_ESM.tif]
