# Supplementary material for: SGK2 promotes prostate cancer metastasis by inhibiting ferroptosis via upregulating GPX4
Source: Cell Death Dis. 2023 Jan 31;14(1):74. doi: 10.1038/s41419-023-05614-5 (PMC9889330; doi:10.1038/s41419-023-05614-5)

Figure 1E

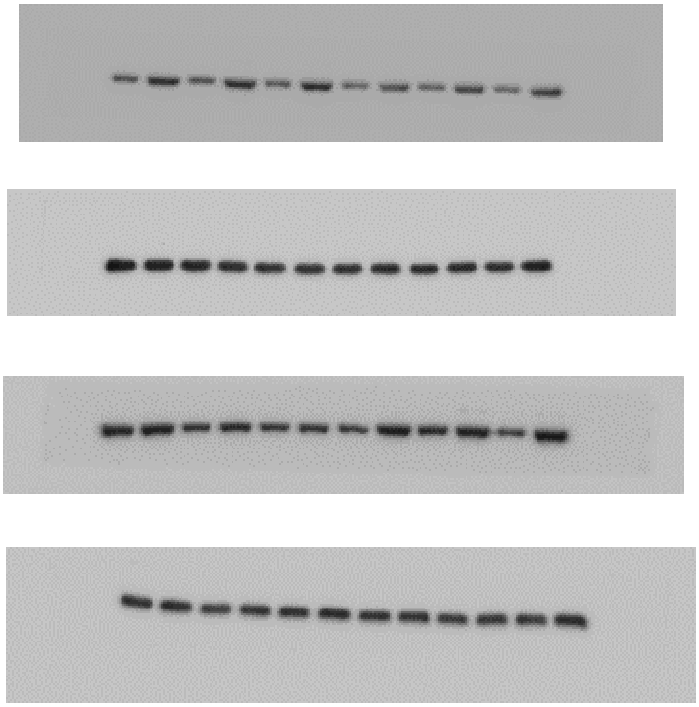

Figure 3A

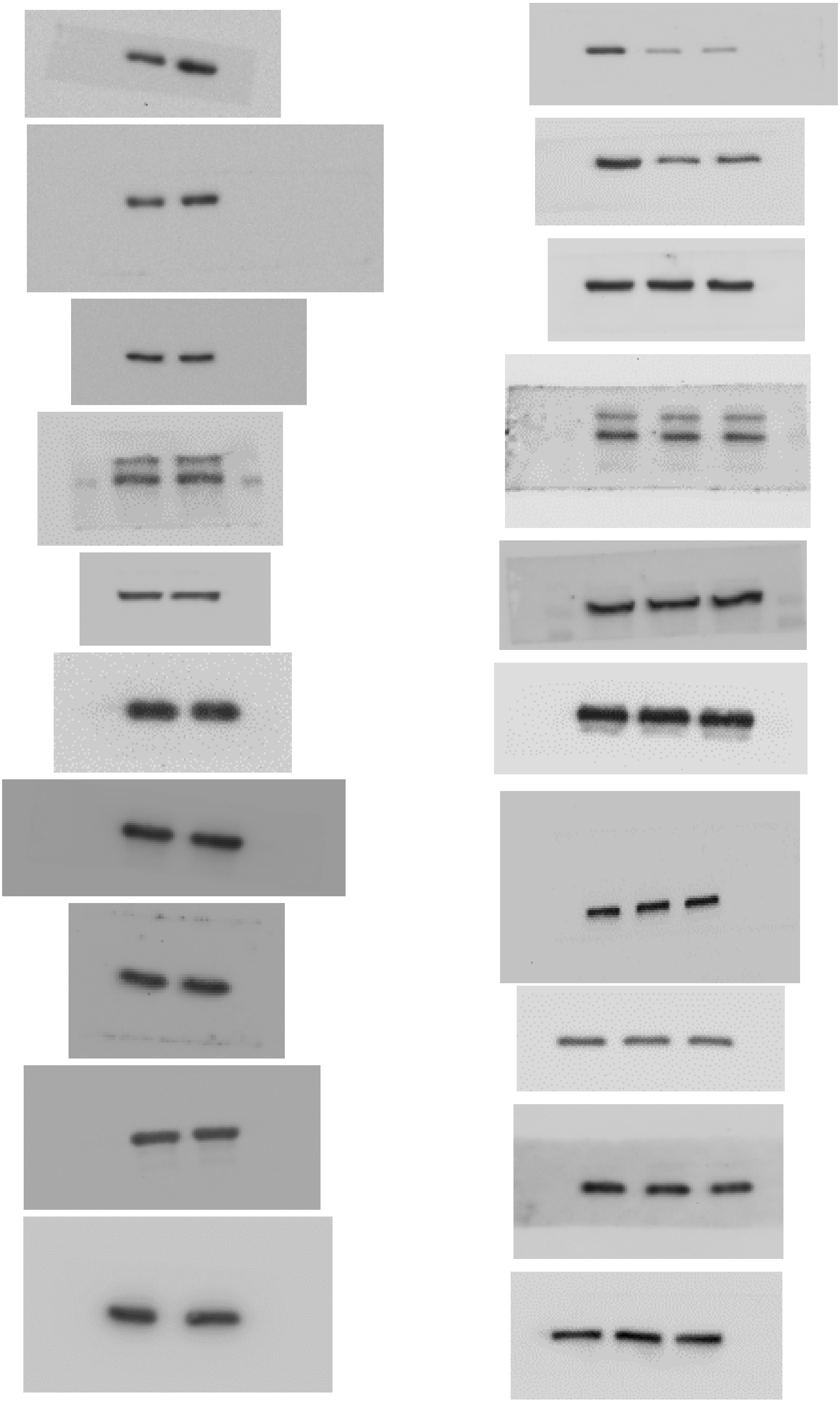

Figure 3B

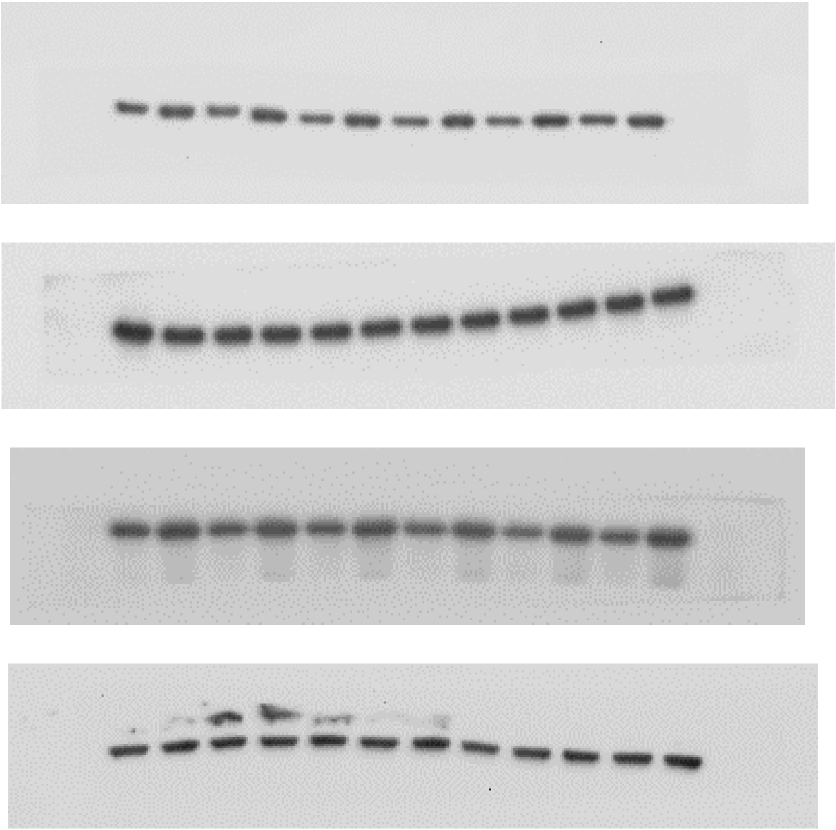

Figure 3E

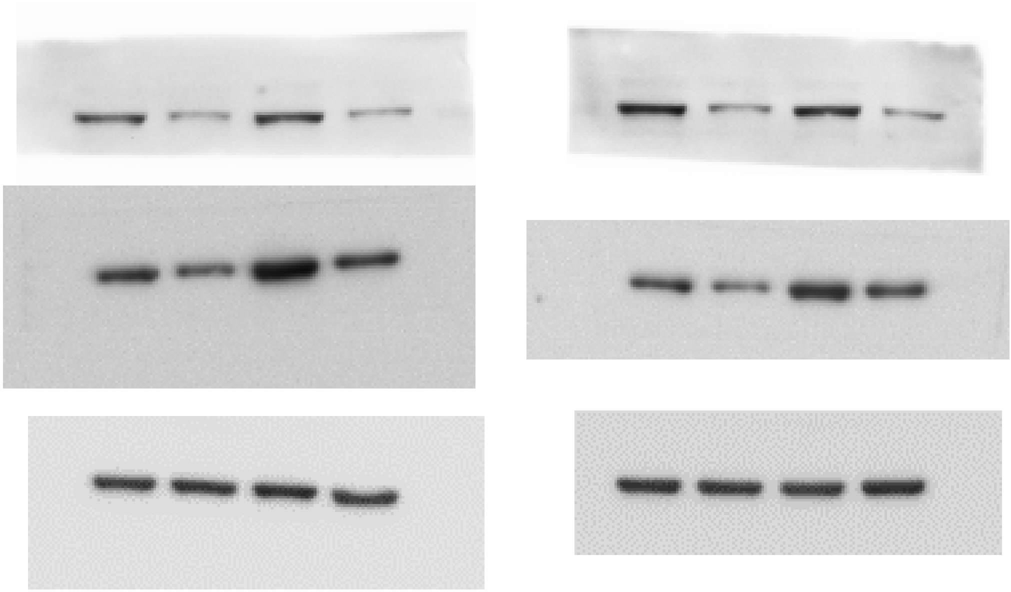

Figure 5A

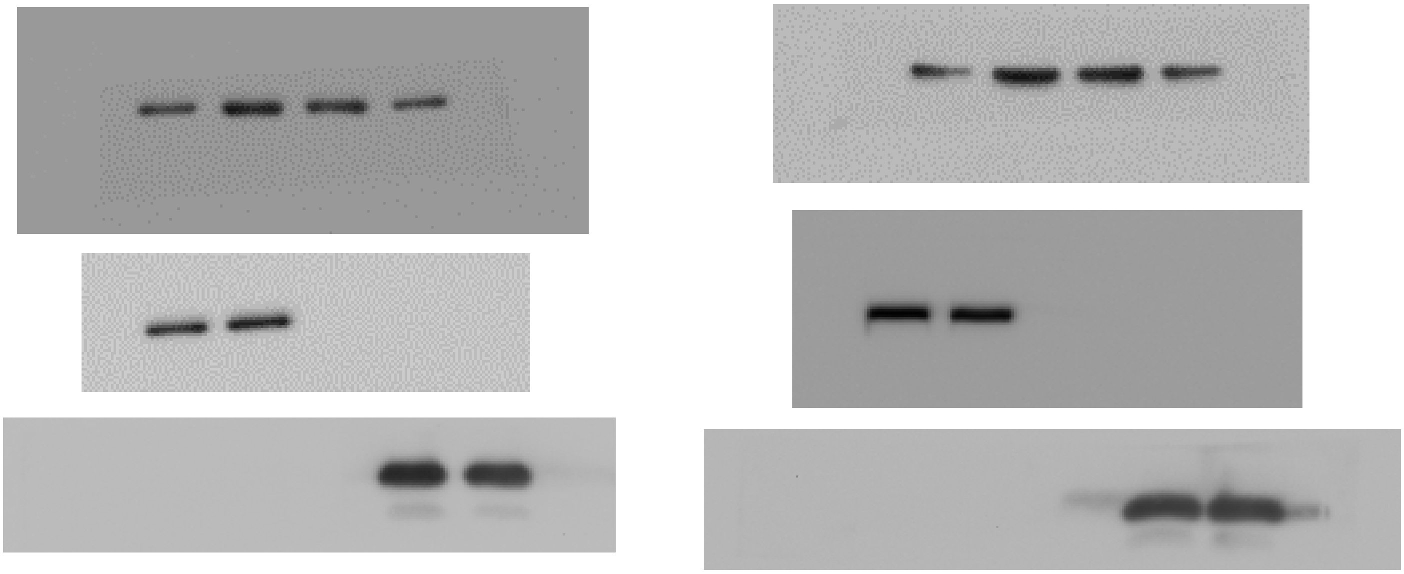

Figure 5B

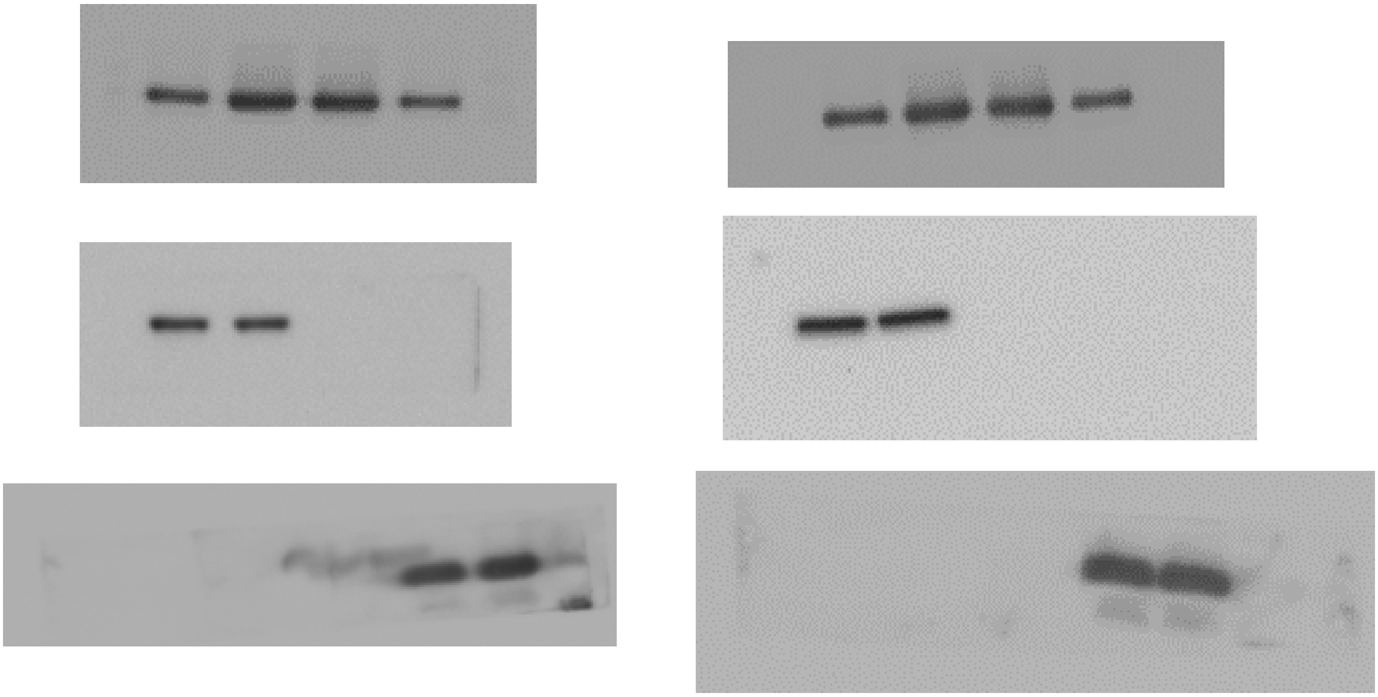

Figure 5I

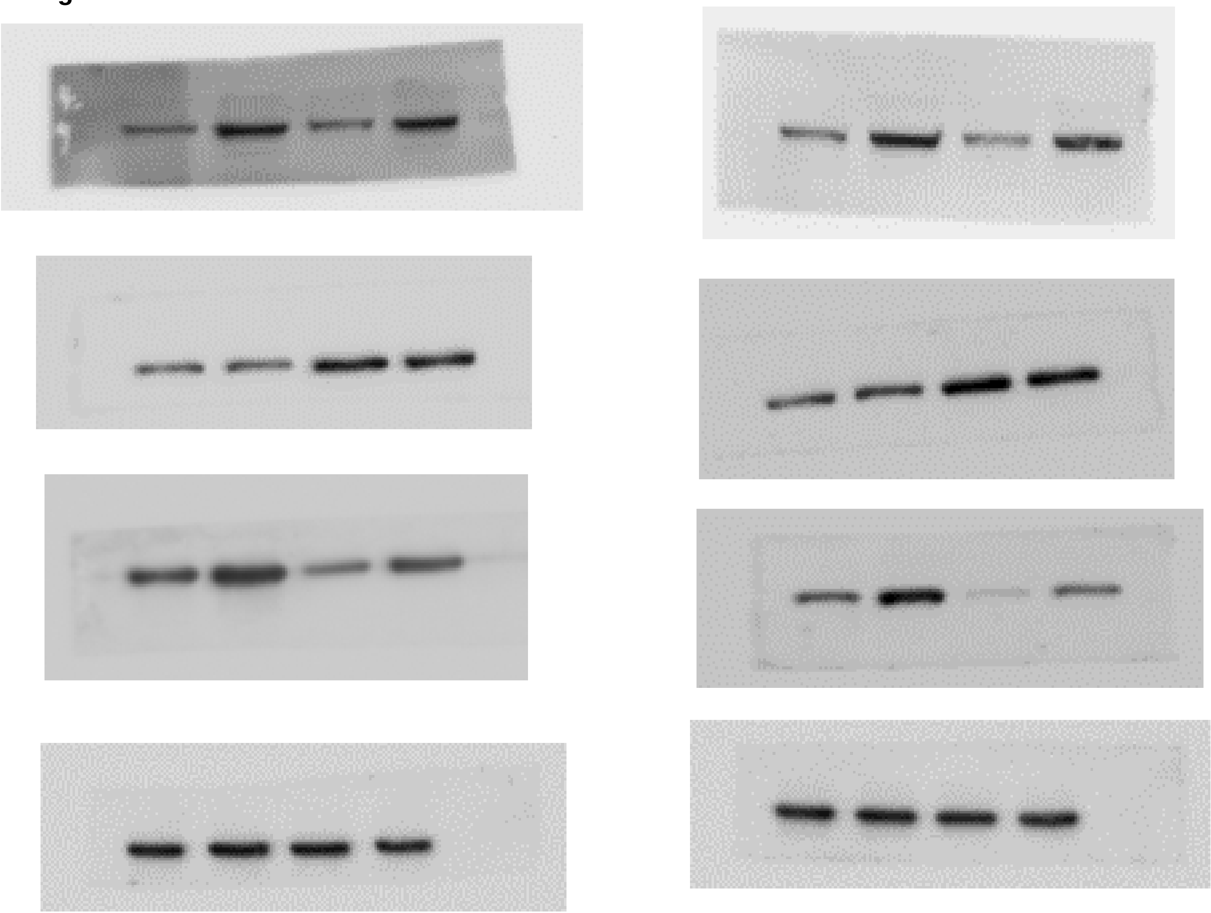

Figure 5J

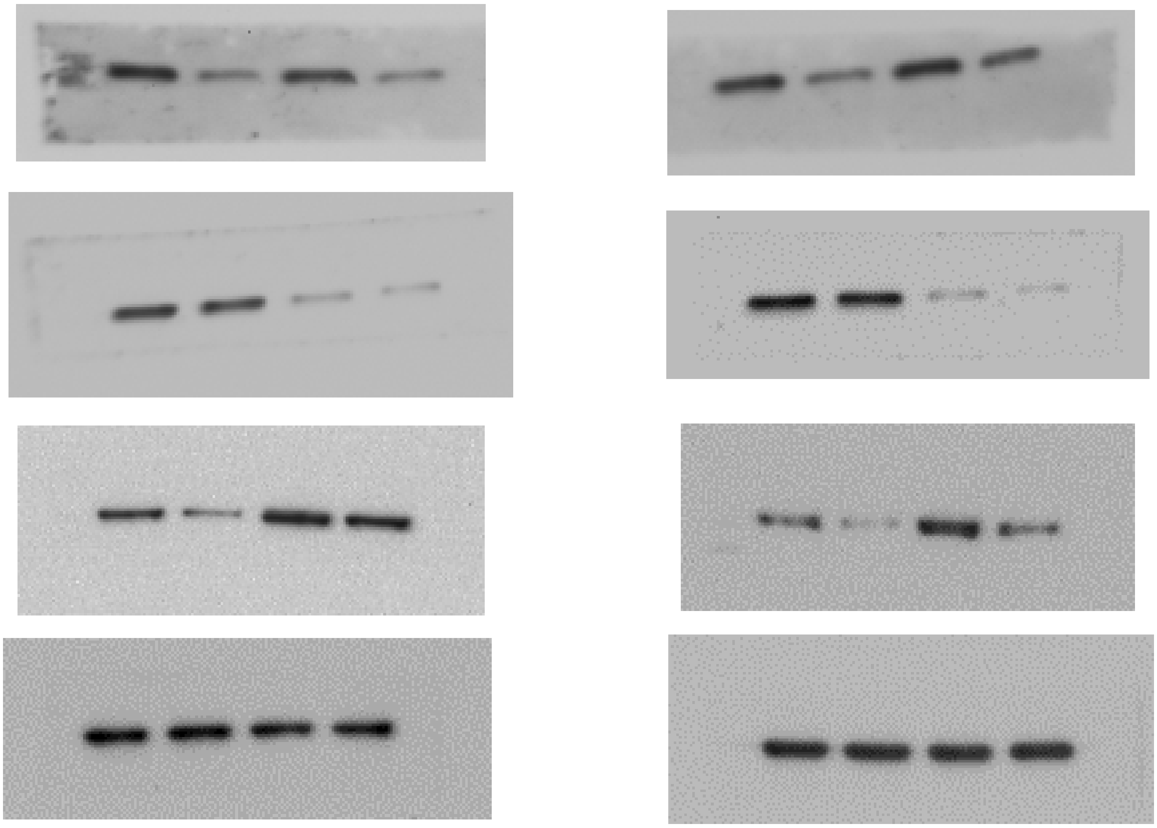

Figure 6B

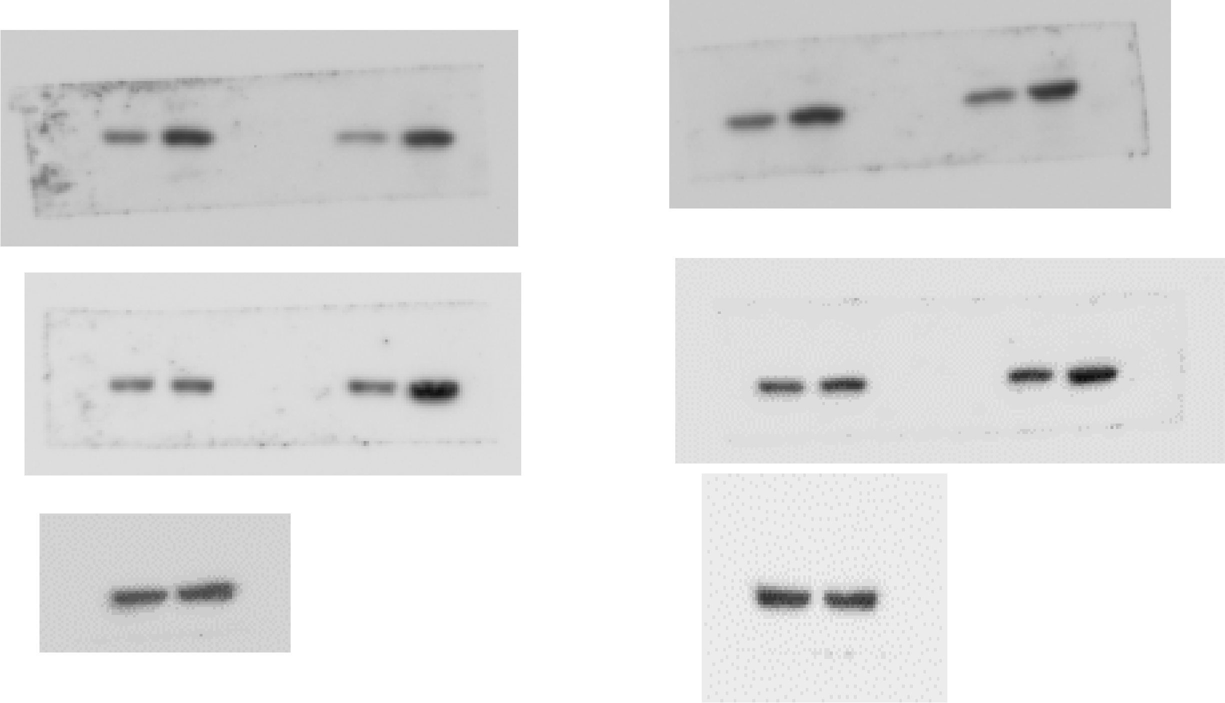

Figure 6C

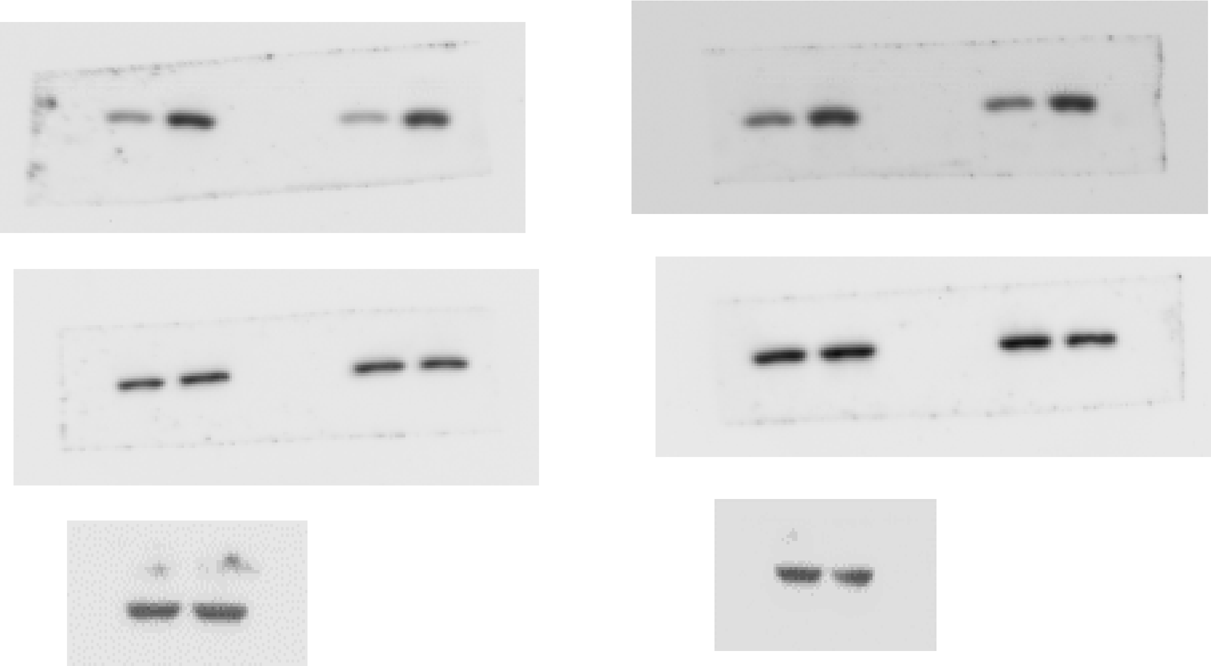

Figure 6D

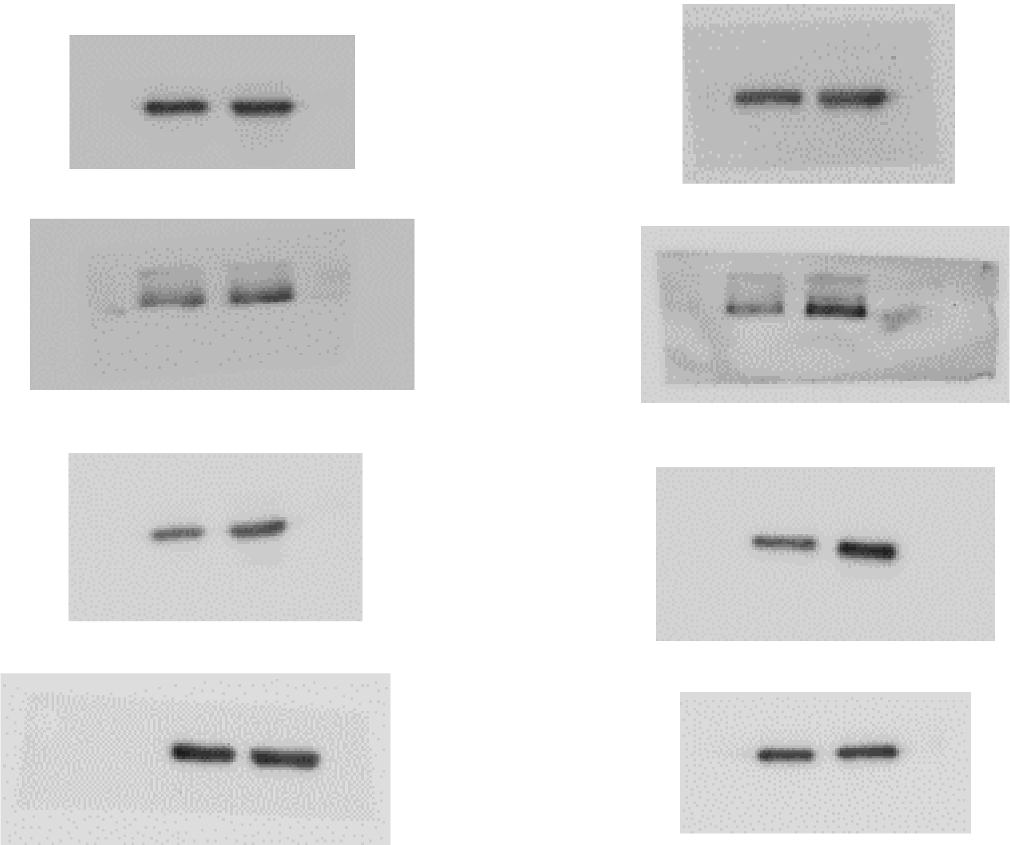

Figure 6F

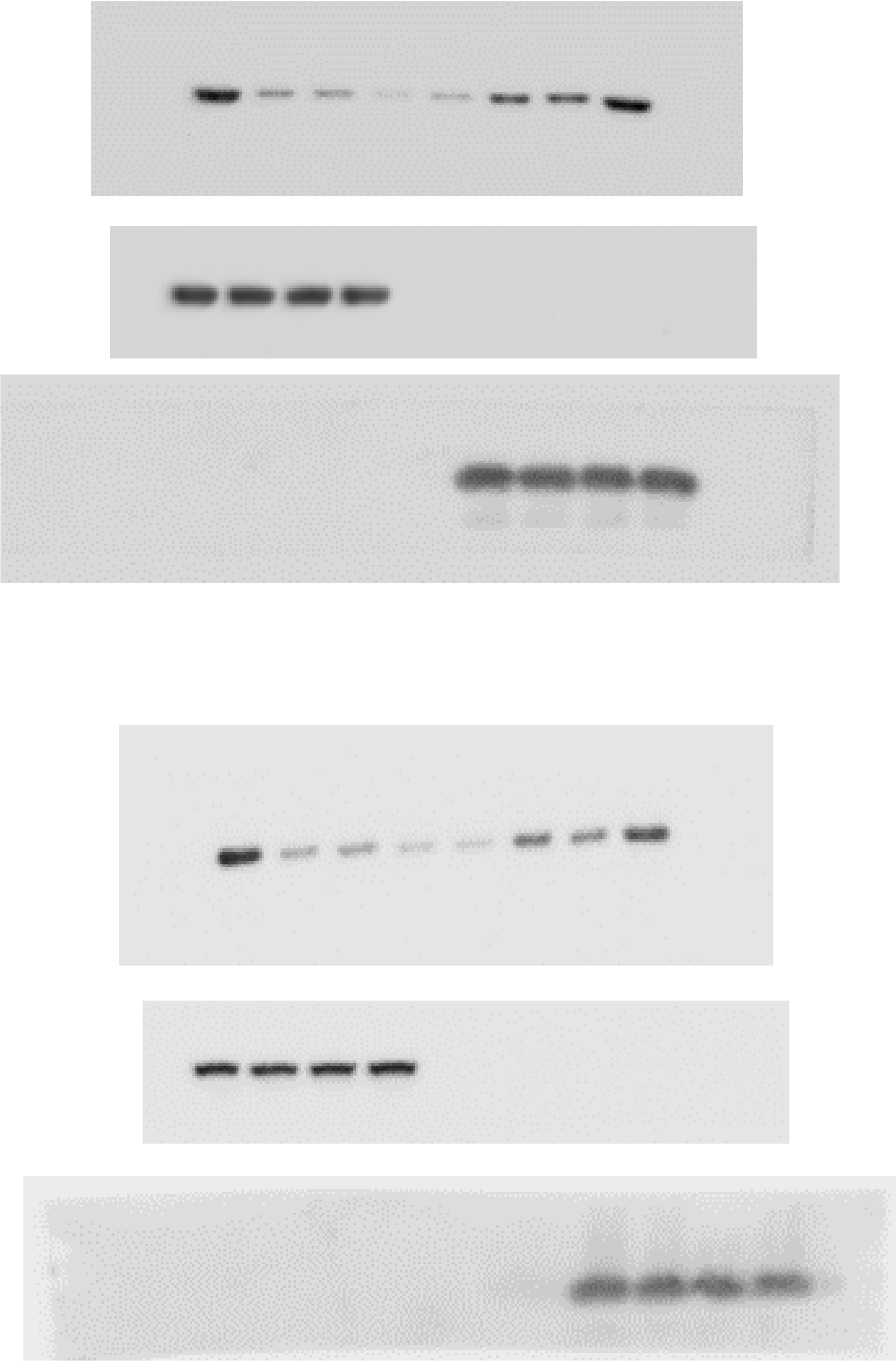

Figure 6G

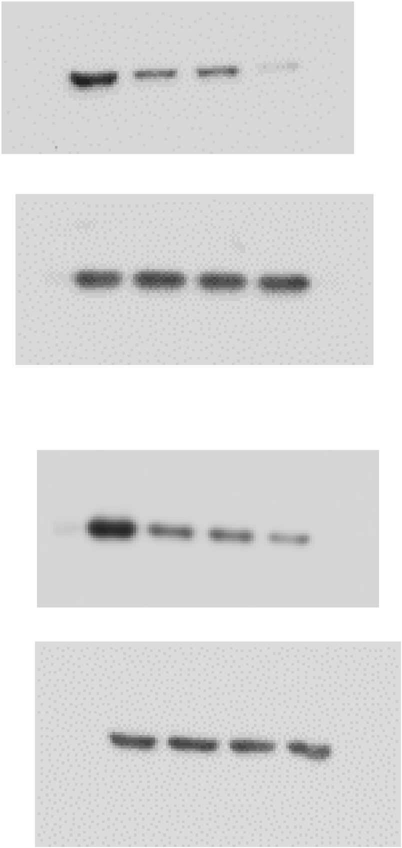

Figure 6J

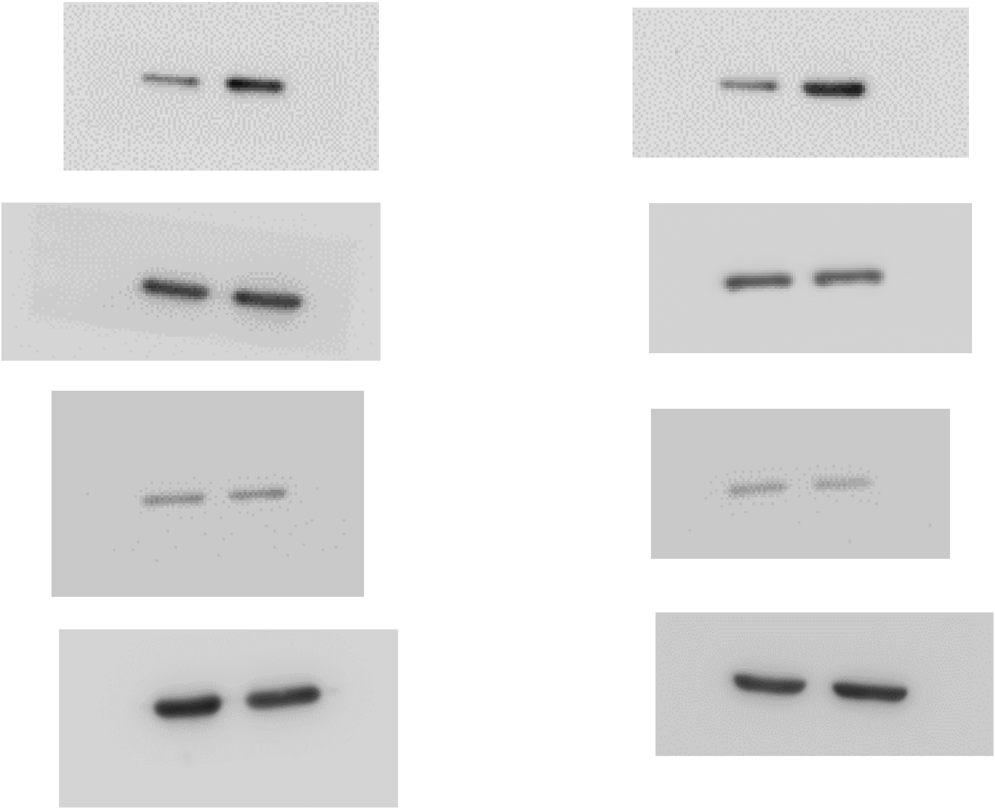

Supplementary Figure S3A

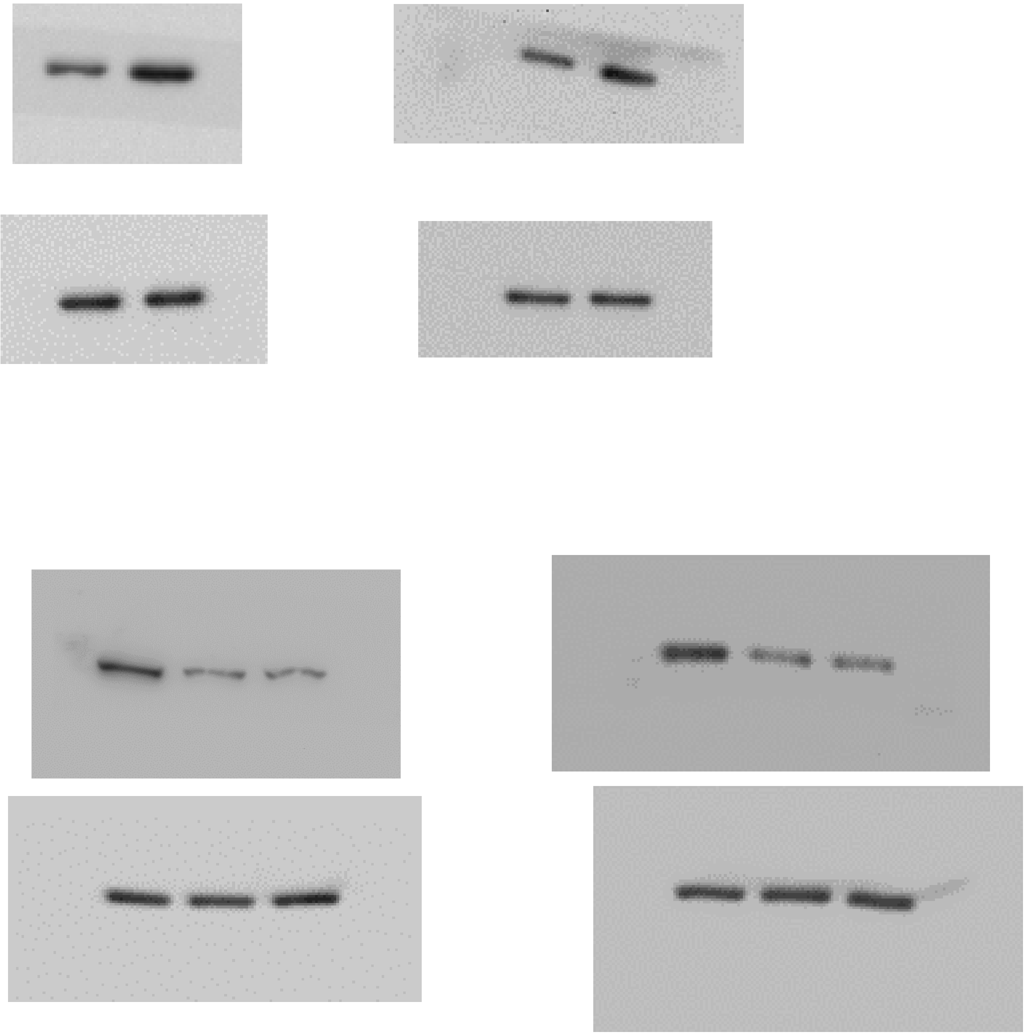

Supplementary Figure S5B

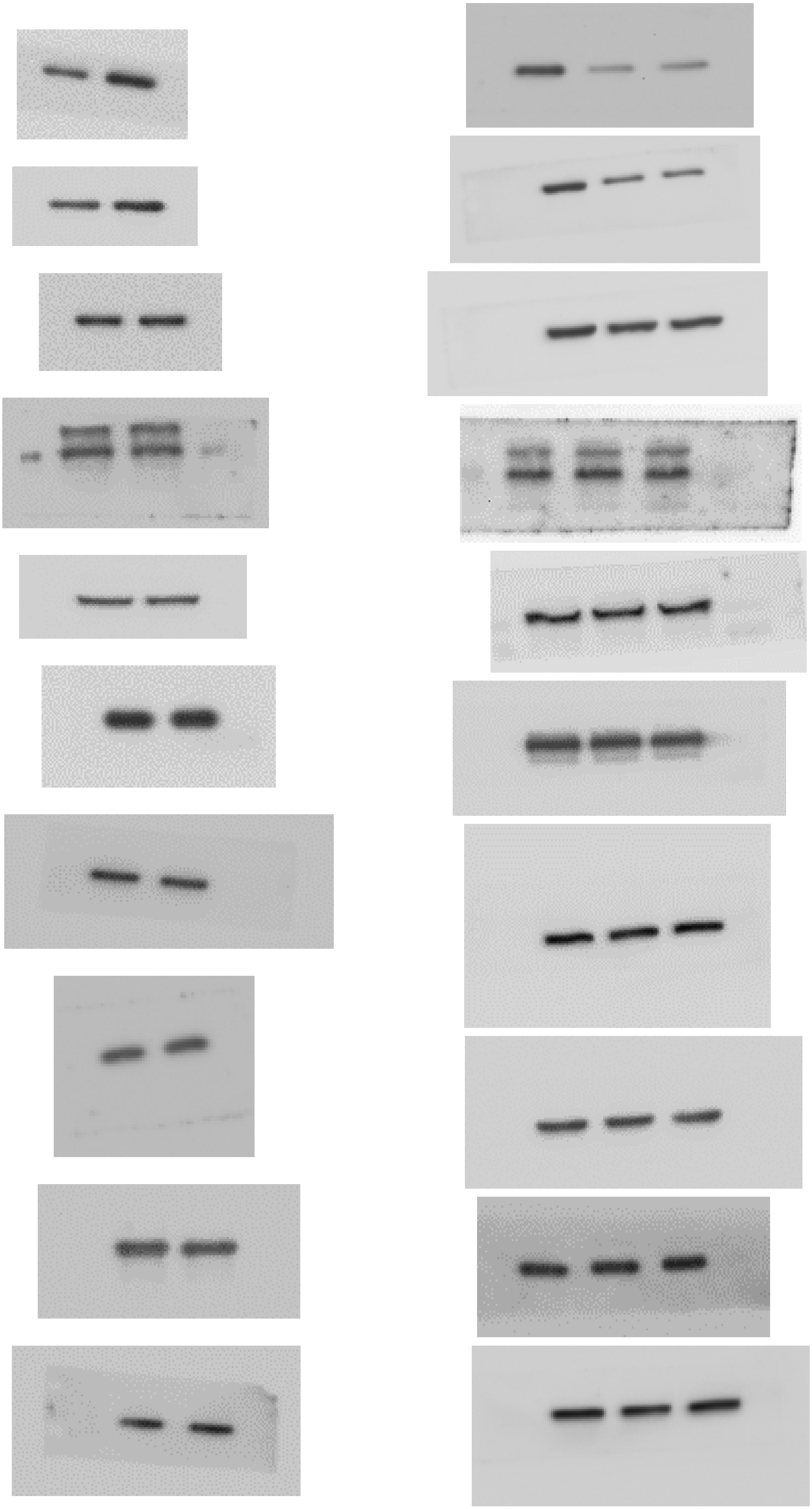

Supplementary Figure S7A

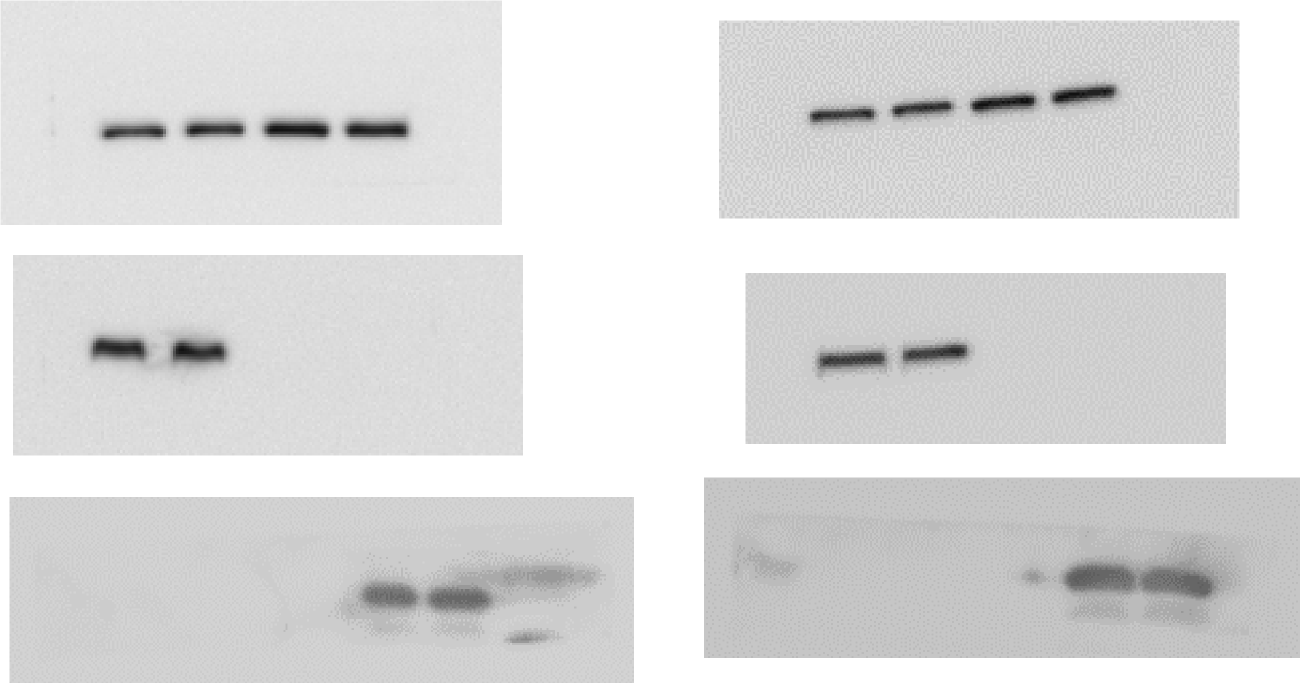

Supplementary Figure S7B

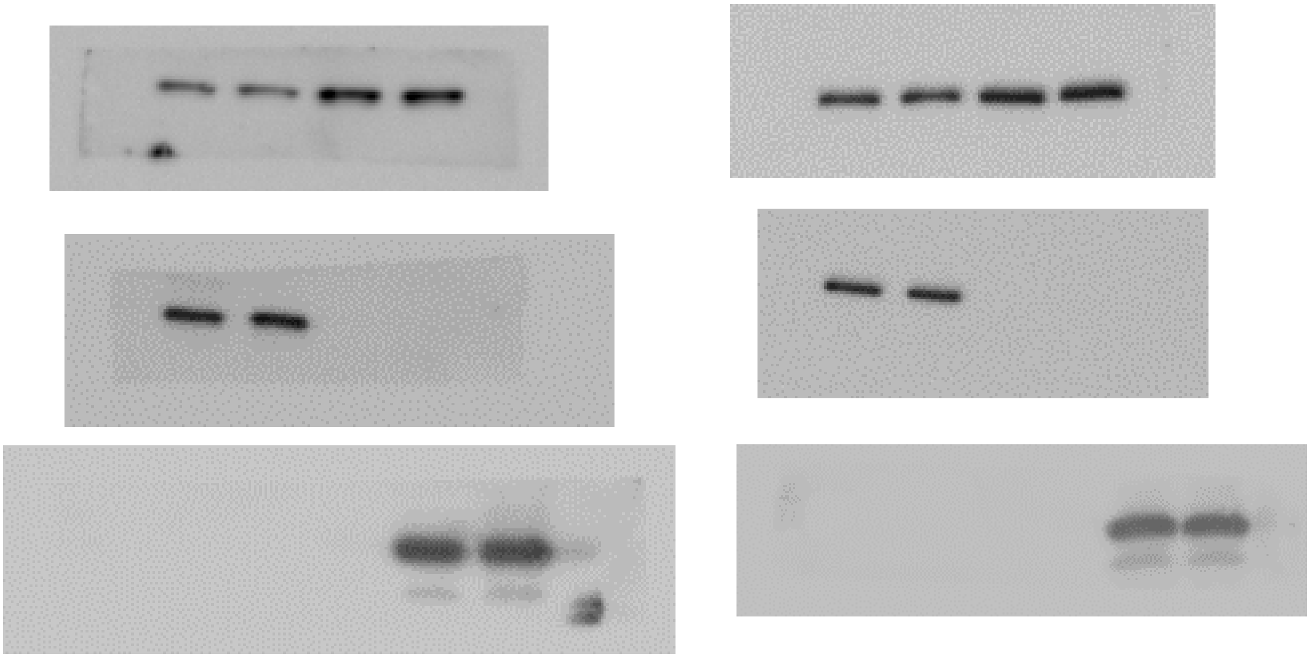

Supplementary Figure S8A

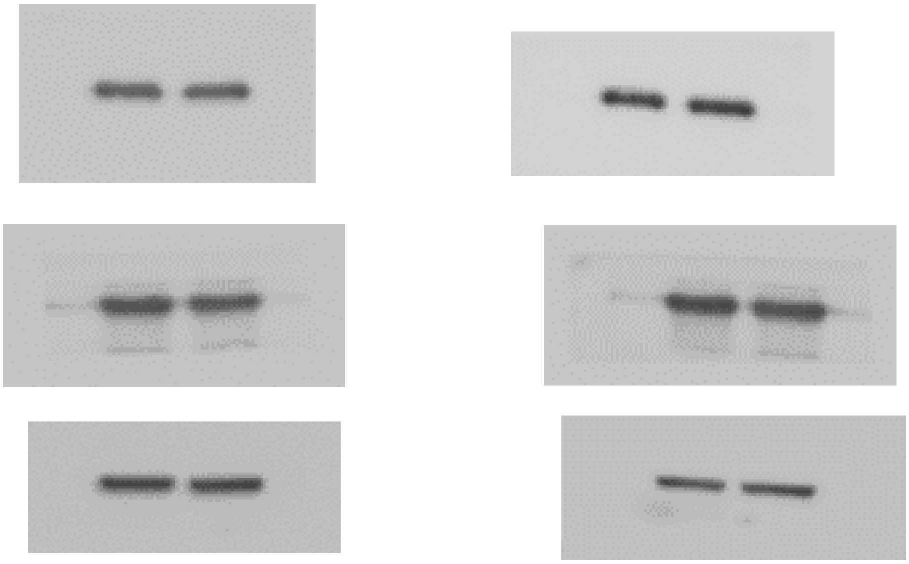

Supplementary Figure S8B

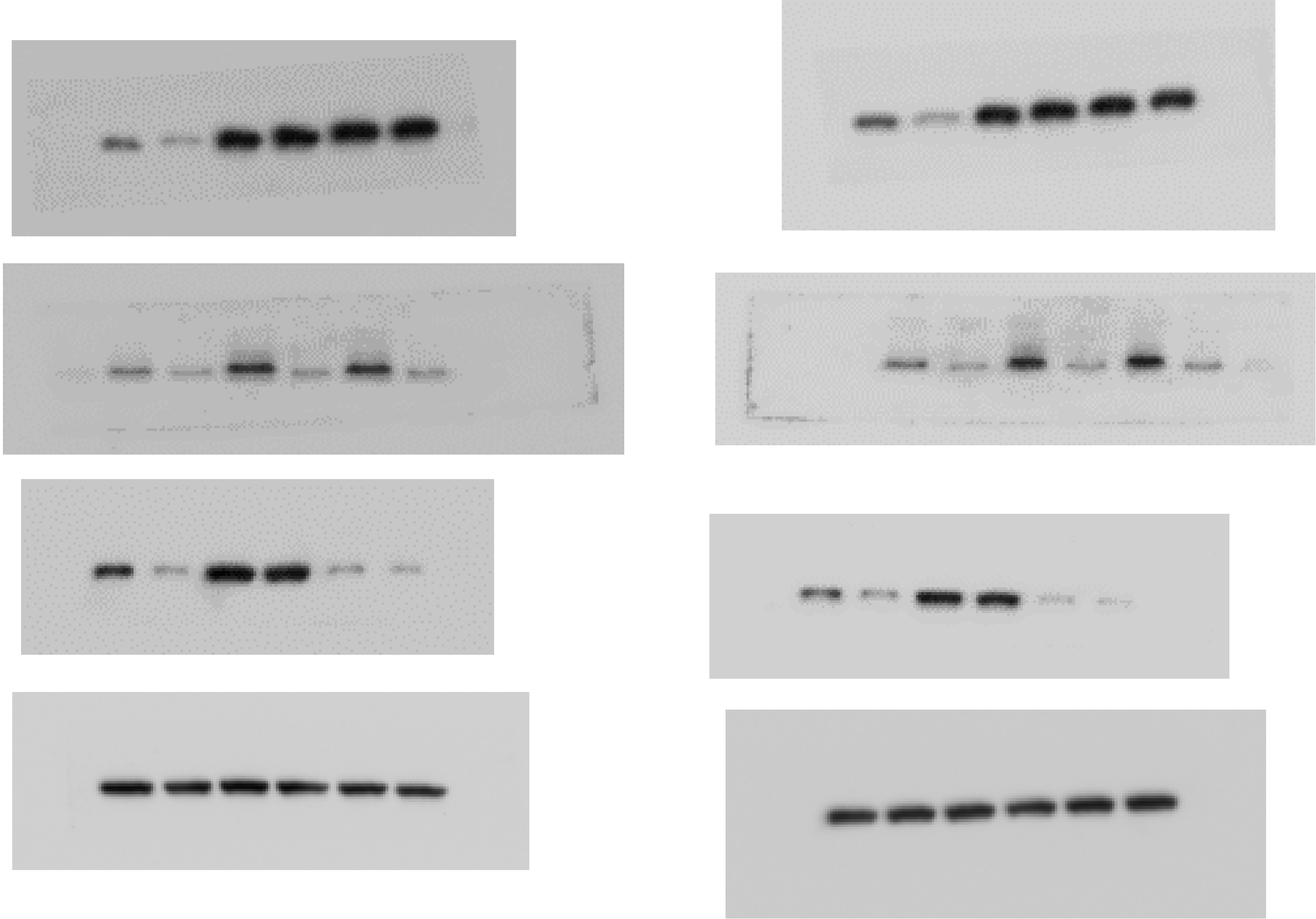

Supplement: Supplementary file 16 — Original western blots [file 41419_2023_5614_MOESM16_ESM.pdf]
